# *In silico* oncology: a mechanistic multiscale model of clinical prostate cancer response to external radiation therapy as the core of a digital (virtual) twin. Sensitivity analysis and a clinical adaptation approach

**DOI:** 10.3389/fphys.2025.1434739

**Published:** 2025-02-24

**Authors:** Georgios Stamatakos, Eleni Kolokotroni, Foteini Panagiotidou, Stamatia Tsampa, Christos Kyroudis, Simon Spohn, Anca-Ligia Grosu, Dimos Baltas, Constantinos Zamboglou, Ilias Sachpazidis

**Affiliations:** ^1^ In silico Oncology and In silico Medicine Group, Institute of Communication and Computer Systems, School of Electrical and Computer Engineering, National Technical University of Athens, Athens, Greece; ^2^ Department of Radiation Oncology, University Medical Center Freiburg, Faculty of Medicine, University of Freiburg, Freiburg, Germany; ^3^ German Cancer Consortium (DKTK), Partner Site Freiburg, Heidelberg, Germany; ^4^ Berta-Ottenstein-Programme, Faculty of Medicine, University of Freiburg, Freiburg, Germany; ^5^ Division of Medical Physics, Department of Radiation Oncology, University Medical Center Freiburg, Faculty of Medicine, University of Freiburg, Freiburg, Germany; ^6^ German Oncology Center, European University Cyprus, Limassol, Cyprus

**Keywords:** cancer, prostate cancer, radiation therapy, multiscale modeling, *in silico* oncology, digital twin, virtual twin, *in silico* medicine

## Abstract

**Introduction:**

Prostate cancer (PCa) is the most frequent diagnosed malignancy in male patients in Europe and radiation therapy (RT) is a main treatment option. However, current RT concepts for PCa have an imminent need to be rectified in order to modify the radiotherapeutic strategy by considering (i) the personal PCa biology in terms of radio resistance and (ii) the individual preferences of each patient.

**Methods:**

To this end, a mechanistic multiscale model of prostate tumor response to external radiotherapeutic schemes, based on a discrete entity and discrete event simulation approach has been developed. Following technical verification, an adaptation to clinical data approach is delineated. Multiscale data has been provided by the University of Freiburg. Additionally, a sensitivity analysis has been performed.

**Results:**

The impact of model parameters such as cell cycle duration, dormant phase duration, apoptosis rate of living and progenitor cells, fraction of dormant stem and progenitor cells that reenter cell cycle, number of mitoses performed by progenitor cells before becoming differentiated, fraction of stem cells that perform symmetric division, fraction of cells entering the dormant phase following mitosis, alpha and beta parameters of the linear-quadratic model and oxygen enhancement ratio has been studied. The model has been shown to be particularly sensitive to the apoptosis rate of living stem and progenitor cells, the fraction of dormant stem and progenitor cells that reenter cell cycle, the fraction of stem cells that perform symmetric division and the fraction of cells entering the dormant phase following mitosis. A qualitative agreement of the model behavior with experimental and clinical knowledge has set the basis for the next steps towards its thorough clinical validation and its eventual certification and clinical translation. The paper showcases the feasibility, the fundamental design and the qualitative behavior of the model in the context of *in silico* experimentation.

**Discussion:**

Further data is being collected in order to enhance the model parametrization and conduct extensive clinical validation. The envisaged digital twin or OncoSimulator, a concept and technologically integrated system that our team has previously developed for other cancer types, is expected to support both patient personalized treatment and *in silico* clinical trials.

## 1 Introduction

*In silico* medicine (ISM) aims to support disease prevention, diagnosis and prognosis, patient-individualized optimization of therapeutic treatment and clinical trials by conducting *in silico* experiments, i.e., experiments on a computer. From a historical perspective, the domain of *in silico* radiation oncology, which is addressed by the present paper, has proved the first paradigm of broader ISM. The formulation of *in silico* radiation oncology has also served as the historic founding landmark of ISM (Stamatakos et al., 2001; Stamatakos et al., 2002; The Yuan, 2022; The Yuan, 2023). Since its founding in 2002, ISM has progressed fast so as to become the focal point and a key objective of several academic, industrial, and regulatory initiatives and societies such as the Virtual Physiological Human Institute (VPH Institute, 2024b) and the Avicenna Alliance–Association for Predictive Medicine (Avicenna Alliance–Association for Predictive Medicine, 2024). Mechanistic multiscale modelling (Deisboeck and Stamatakos, 2011; Kolokotroni et al., 2024), eventually hybridized with artificial intelligence and/or advanced statistics, frequently serves as the core of digital (or otherwise known as virtual) twins in medicine. Digital twins are an emerging technology that builds on *in silico* representations of an individual or parts of it that dynamically reflect their multiscale biological, physiological, pathological and medical status over time (Bruynseels et al., 2018; VanDerHorn and Mahadevan, 2021; Hernandez-Boussard et al., 2021). A paradigm of digital twin is the OncoSimulator—the first digital twin in oncology and beyond (VPH Institute, 2024a; Stamatakos et al., 2007; Stamatakos, 2011; Stamatakos et al., 2014; European Commission, Cordis, EU Research Results, 2024; Kolokotroni et al., 2024). The primary aim of the OncoSimulator is to optimize cancer treatment and make it more patient specific by conducting *in silico* experiments. Additionally, it constitutes a platform for investigating the natural phenomenon of cancer, supporting the design and interpretation of clinical trials and training doctors, researchers and interested patients alike. The importance and the great clinical potential of cancer digital twins has been recently revisited (Hernandez-Boussard et al., 2021; Stahlberg et al., 2022). The work presented here serves as the core of the prostate cancer OncoSimulator.

Prostate cancer (PCa) is the most frequently diagnosed malignancy in male patients in Europe and radiation therapy (RT) is a main treatment option. However, current RT concepts for PCa have an imminent need to be rectified in order to modify the radiotherapeutic strategy by considering (i) the personal PCa biology in terms of radio resistance and (ii) the individual preferences of each patient. To this end, a number of mechanistic computational models of prostate cancer growth (Lorenzo et al., 2016; Phan et al., 2020) and relapse (Stura et al., 2016) with miscellaneous proposed clinical applications have appeared in the literature. Artificial intelligence has also been adopted to improve prostate cancer diagnosis and prognosis (Yi et al., 2022; John et al., 2021). However, due to the multiplicity of prostate cancer treatment approaches, no single computational model appears to be applicable to all treatment options.

In this context, a clinically-oriented, mechanistic, multiscale, spatiotemporal simulation model of prostate tumor free growth and response to radiotherapy is presented and investigated. It is to be noted that free growth is an approximation to restricted growth of a tumor within the soft organ of the prostate, where mechanical deformations are allowed to take place to a certain extent. It is also noted that the term *soft* is used here in the sense of tissue other than bone or cartilage. The model core stems from previous work of the *In silico* Oncology and *In silico* Medicine Group, National Technical University of Athens (Stamatakos, 2024). The model addresses tumors well beyond their initiation phase and aims at simulating their spatiotemporal evolution. It has been designed to incorporate patient-specific data such as imaging-based (e.g., MRI), histopathological (e.g., Gleason score, apoptotic index), molecular (e.g., Ki-67) and treatment data (e.g., radiotherapy dose per session, number of fractions, intervals between fractions). The proposed model is primarily based on a discrete entity and discrete event simulation approach (Stamatakos et al., 2010; Kolokotroni et al., 2016; Kyroudis et al., 2019).

Following technical verification, an adaptation to clinical data approach is outlined through the utilization of two patients data and an initial exploration of the model’s potential is delineated. The clinical adaptation approach is meant to serve as a proof-of-concept procedure, aiming at demonstrating the feasibility of using cancer modeling in clinical practice in order to optimize radiotherapy treatment. Real data has been provided by the University of Freiburg, within the framework of the European Commission (EC) supported project PersoRad (ERAPERMED2019-299).

Additionally, a parametric and a sensitivity analysis, which have revealed the impact of particular model parameters on the overall model behavior, have been performed. This constitutes one of the first steps in increasing the robustness of the model. Such a study is essential, *inter alia*, for the identification of plausible parameter value ranges in order to guarantee a biologically relevant virtual tumor behavior. Furthermore, aspects of the interplay and possible interdependencies of the biological mechanisms modeled, which often cannot be grasped intuitively, can be enlightened and experimental biological observations can be deciphered. Finally, sensitivity analysis helps to explore the model’s behavior in relation to the value of each chosen input parameter, with the primary aim to deepen and advance quantification of our understanding of tumor response to treatment. Indicative aspects of the model addressed by the sensitivity analysis include the temporal evolution of the following quantities: tumor volume reduction, fraction of tumor stem cells, fraction of terminally differentiated tumor cells and fraction of dead tumor cells. A qualitative agreement of the proposed model behavior with published experimental and clinical knowledge and data for two patients has set up the basis for the next steps towards its thorough clinical validation and its eventual certification and clinical translation. It is pointed out, however, that this paper represents initial work. Its goal is to demonstrate the feasibility and the fundamental design and behavior of the model as the key component of a digital twin. Further multiscale clinical data is being collected by our team in order to enhance the model parametrization and implement a formal clinical validation of the model. The corresponding digital twin or OncoSimulator, a concept and a technologically integrated system that our team has previously developed and validated for other cancer types (Stamatakos et al., 2014; European Commission, Cordis, EU Research Results, 2024), is expected to serve both as a clinical decision support system for patient individualized treatment and as a platform for *in silico* clinical trials.

## 2 Materials and methods

The model presented is based on an algorithmic description of discrete events (such as the change of the cell cycle phase from phase G2 to mitosis) which happen in discrete entities (such as a tumor cell). Space and time are discretized and all state transitions are implemented algorithmically within the framework of a discretizing mesh superimposed onto the anatomic region of interest (Stamatakos, 2011; Kolokotroni et al., 2016). Therefore, no differential equations are employed in this approach. The discrete algorithmic application of biological and physical laws and rules generates the spatiotemporal evolution of the tumor as well as the time course of the states of all cell populations considered. Specific information contained in the imaging slices (e.g., MRI slices) considered, such as regions with high and low oxygenation, are exploited by appropriately marking the corresponding regions of the 3D reconstruction of the tumor and by changing the values of pertinent parameters such as the probability of a dormant cell to re-enter the cell cycle (Stamatakos et al., 2002).

In this section as a first initiating step, an algorithmic and graphical description of the tumor growth and response to treatment model is outlined. This includes the description of the involved major biological processes, including the cytokinetic model as well as the model implementation choices. Subsequently, the full simulation procedure is outlined.

### 2.1 The tumor growth and response to treatment component model—model description

#### 2.1.1 The cytokinetic model—biological processes considered

Tumors usually consist of a cluster of heterogeneous cell populations with variable proliferative potential. Experimental observations have indicated that tumor sustenance is attributed to the so-called cancer stem cells (Bjerkvig et al., 2005; Gupta et al., 2009), a cell population exhibiting stem cell-like properties, such as unlimited self-renewal and differentiation capacity. Moreover, tumors, not only among different but also within the same tumor type, are characterized by variable differentiated cell composition. The model takes into account cells of distinct mitotic potential and supports the simulation of tumors of different differentiation degree as reflected in the relative percentage of proliferating and differentiated cells.

**FIGURE 1:**
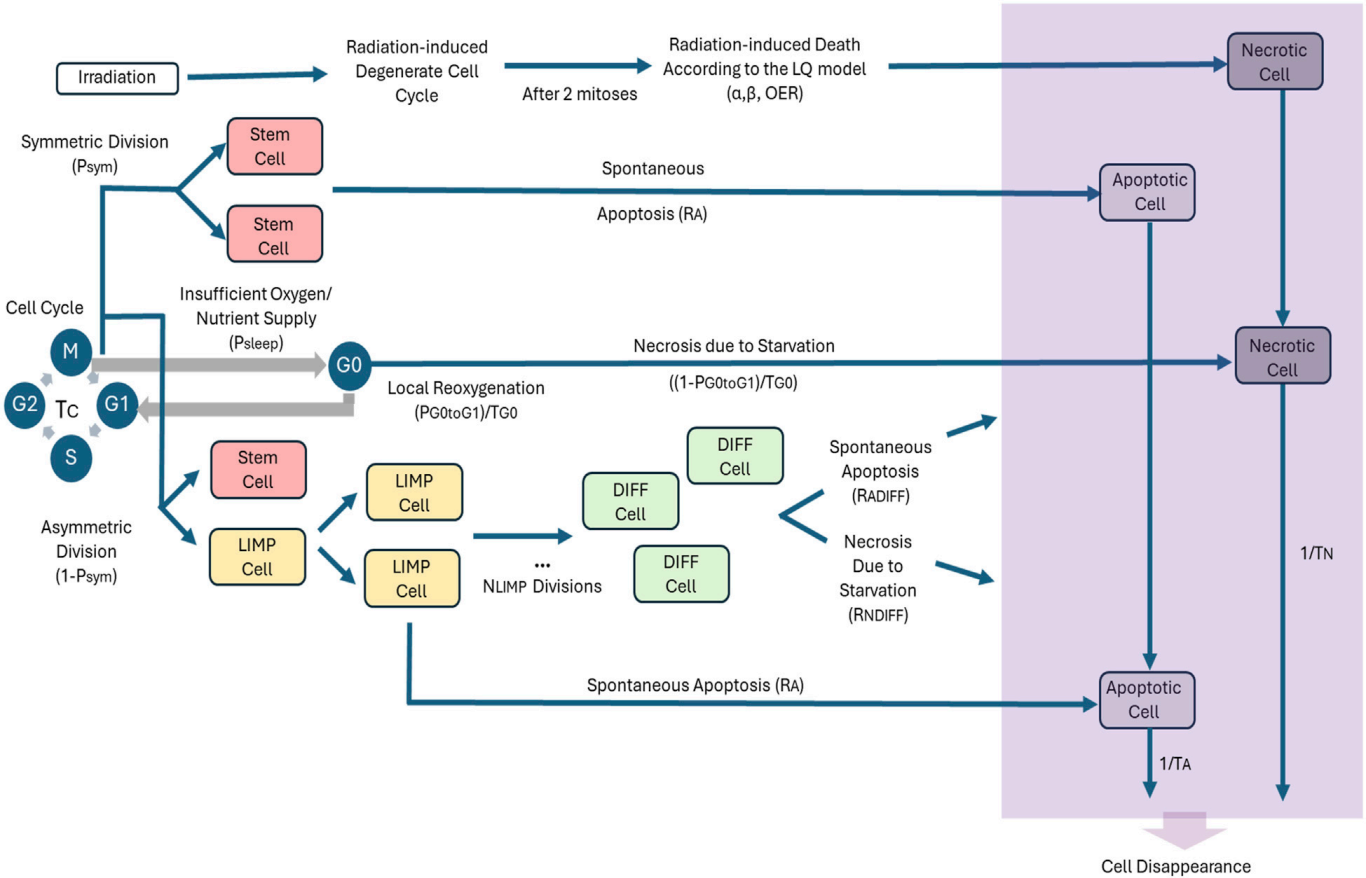
Generic cytokinetic model (cell category/phase transition diagram) for tumor response to radiotherapy. Symbols and abbreviations: LIMP: LImited Mitotic Potential tumor (cell), also called committed or restricted progenitor cell. DIFF: terminally DIFFerentiated tumor (cell). G1: Gap 1 cell cycle phase. S: DNA synthesis phase. G2: Gap 2 phase. M: Mitosis phase. G0: dormant, resting phase. OER: Oxygen Enhancement Ratio, α: alpha parameter of the Linear-Quadratic (LQ) model, β: beta parameter of the LQ model, P_sym_: probability of a stem cell to perform symmetric division (or equivalently, fraction of stem cells that perform symmetric division), R_A_: apoptosis rate of living stem and LIMP tumor cells (fraction of cells dying through apoptosis per unit time), T_c_: cell cycle duration, P_sleep_: fraction of cells entering the G0 phase following mitosis, P_G0toG1_: fraction of dormant (stem and LIMP or Limited Mitotic Potential) cells that re-enter cell cycle, T_G0_: G0 (dormant phase) duration, i.e., time interval before a dormant cell enters necrosis, R_NDIFF_: necrosis rate of differentiated tumor cells, R_ADIFF_: apoptosis rate of differentiated tumor cells, T_N_: Time needed for both necrosis to be completed and its lysis products to be removed from the tumor, T_A_: Time needed for both apoptosis to be completed and its products to be removed from the tumor.

The adopted cytokinetic model shown in Figure 1 incorporates the biological mechanisms of cell cycling, quiescence, recruitment, differentiation and loss via apoptosis (either spontaneous or treatment-induced) and necrosis (starvation-induced). Tumor sustenance is attributed to the presence of the cancer stem cells, which have the ability to preserve their own population. Two types of cancer stem cell division are possible: symmetric and asymmetric. Usually, symmetric cell division produces two daughter cells of the same fate, either stem cells or more differentiated progenies; while asymmetric cell division produces daughter cells of different fates. (Chao et al., 2024; Majumdar and Liu, 2020). As a first approximation, in this paper a symmetric cancer stem cell division gives rise to two daughter cells, both with a cancer stem cell fate, whereas an asymmetric cancer stem cell division gives rise to one daughter cell with cancer stem cell fate and one daughter cell with limited mitotic potential or committed progenitor cancer cell fate that follows the path towards differentiation. Specifically, the following five categories of cancer cells are considered in the model:

i. Stem cells: cells able to perform unlimited number of divisions.
ii. Limited mitotic potential (LIMP) or restricted/committed progenitor cells: cells able to perform a limited number of divisions before becoming terminally differentiated.
iii. Differentiated cells: terminally differentiated cells with no mitotic capacity.
iv. Apoptotic cells: cells that have died through apoptosis.
v. Necrotic cells: cells that have died through necrosis.

Stem, LIMP and differentiated cells constitute three categories with distinct mitotic potential.

A proliferating tumor cell (stem or LIMP) goes through the four phases of the cell cycle: gap 1 (G1) phase, DNA synthesis (S) phase, gap 2 (G2) phase and mitosis (M) phase. After the completion of mitosis, a fraction of newborn cells will enter the dormant phase, due to insufficient nutrient supply and oxygenation, whereas the rest will continue to cycle. Under conditions of lack of nutrients, dormant cells are assumed to survive only for a limited time length. After the expiration of this time, dormant cells die through necrosis, unless the local conditions of nutrient and oxygen supply have been reinstated, allowing the re-entering of the dormant cell into the active cell cycle. Any cell may die through spontaneous apoptosis. Differentiated cells may die through apoptosis or necrosis. Table 1 lists the model parameters and the corresponding biological mechanisms as described above.

**TABLE 1:** Code input parameters.

Parameter symbol	Description	Unit	Value range
Cell phase durations			
T_c_ [class^a^]	Cell cycle duration	hour	—
T_G0_ [class^a^]	G0 (dormant phase) duration, i.e., time interval before a dormant cell enters necrosis	hour	—
T_N_ [region^b^]	Time needed for both necrosis to be completed and its lysis products to be removed from the tumor	hour	—
T_A_ [region^b^]	Time needed for both apoptosis to be completed and its products to be removed from the tumor	hour	—
Cell category/phase transition rates and fractions			
R_A_	Apoptosis rate of living stem and LIMP tumor cells (fraction of cells dying through apoptosis per unit time)	hour^−1^	0–1
R_NDiff_	Necrosis rate of differentiated tumor cells	hour^−1^	0–1
R_ADiff_	Apoptosis rate of differentiated tumor cells	hour^−1^	0–1
P_G0toG1_ [class^a^][region^b^]	Fraction of dormant (stem and LIMP) cells that re-enter cell cycle	—	0–1
P_sleep_ [region^b^]	Fraction of cells entering the G0 phase following mitosis	—	0–1
P_sym_ [region^b^]	Fraction of stem cells that perform symmetric division	—	0–1
Miscellaneous parameters			
Cell density	Number of biological cells normally contained within a unit volume	mm^−3^	10^6^
Voxel dimension	Dimension of voxel/GC edge	mm	1 or 2, depends on tumor size and computing resources
N_LIMP_	Number of mitoses performed by LIMP cells before becoming differentiated	—	≥1
x_dim_, y_dim_, z_dim_	Number of geometrical cells along the *x*, *y*, *z*-axis respectively	—	Depends on tumor size and computing resources
tumor _length	Dimensions of the three tumor axes in mm in the case a triaxial ellipsoidal tumor is considered	—	Depends on tumor imageable characteristics
tumor_breadth			
tumor_width			
necrotic _length	Dimensions of the necrotic region along the three axes in GCs in case a triaxial ellipsoidal tumor is considered	—	Depends on tumor imageable characteristics
necrotic _breadth			
necrotic _width			
Execution time	Execution stop time after initialization	day	—
Spatial evolution?	Will geometric spatial evolution be included in the simulations?	—	0 or 1
	“True”: yes. The functions for tumor differential expansion/shrinkage are enabled		
	“False”: no. The functions for tumor differential expansion/shrinkage are disabled		
margin_percent	Acceptable temporary over-loading or under-loading of each geometrical cell as a fraction of unity	—	0.0–0.5
color_criterion	Minimum percentage of tumor cells that should be dead in order to denote (“paint”) the corresponding geometrical cell as necrotic	—	0.9–0.999
Input image?	True: consider the input image file	—	0 or 1
	False: no input image file		
Image filename	Name of the input image file	—	—
Output directory	Name of the directory where the output files are stored	—	—
mode	The tumor course to be simulated	—	1: Free growth, 2: treatment response
Radiotherapy parameters			
T_radio,adm_ [n]	Time point of *n*th radio administration, n = 1,…	day	Depends on clinical data
α	alpha parameter of the LQ model	Gy^−1^	Literature based
β	beta parameter of the LQ model	Gy^−2^	Literature based
OER	Oxygen Enhancement Ratio	—	Literature based
Cell kill factor	Factor adapting cell killing probability to stem cells	—	0–1

^a^ Defined separately for **stem** and **LIMP**, cells [class: {stem, LIMP}].

^b^ Defined separately for **proliferating** and **necrotic** region [region: {proliferating, necrotic}].

See Section 3.1.1 for specific table parameter value ranges pertinent to the analysis conducted in this article and ranges of other quantities utilized in the process of clinical adaptation of the model.

For a given cancer cell, if it has been decided to enter the cell cycle, it is assumed that there is adequate oxygenation through all cell cycle phases till the completion of mitosis. This is a simplification assumption, that may create a very small/differential local quantization error, which is nevertheless expected by a complex model. Just after completion of mitosis, a new decision for the fate of its two offspring cancer cells is taken. They could either enter the hypoxic dormant phase G0 or enter the cell cycle. Therefore, at any given time point a cell could either reside in the G0 phase due to hypoxia or reside within the cell cycle and be aerobic (well oxygenated).

It should be noted that for practical simplification reasons, the decision as to whether a tumor cell is to proceed to mitosis is taken before the latter enters the cell cycle phase G1. Following the latest mitosis, the two newborn cells re-enter together the cell cycle, in case the oxygenation level in the region they reside is adequate. Otherwise, they enter the dormant G0 phase. If within the time interval T_G0_, oxygenation has not become adequate for cell cycling and division, both cells enter necrosis. In case that imaging data provides spatial information on the oxygenation distribution within the tumor, this along with empirical rules describing the expected time course of the expansion or shrinkage of well oxygenated areas within a tumor (Stamatakos et al., 2002) is taken into account when deciding on the cycling, dormancy or necrosis fate of a tumor cell. Otherwise, a constant mean value of each one of the two pertinent parameters: “fraction of cells entering the G0 phase following mitosis (Psleep) and “fraction of dormant (stem and LIMP) cells that re-enter cell cycle (PG0toG1)” (Table 1) is considered throughout a given simulation. Suitable mean values for the latter are provided by the calibration of the model for different tumor subtypes through the clinical adaptation procedure. The latter approach has been applied to the specific clinical cases addressed by the present paper.

Cell killing by irradiation is described by the Linear-Qadratic (LQ) Model, which is widely used in the pertinent literature (Steel, 2002) (Equation 1):

$$S\left(D\right)=\exp\;\left[-\left(\alpha D+\beta D^{2}\right)\right]$$ (1)

where S(D) is the surviving fraction after a (uniform) dose D (Gy) of radiation to a population of cells and α (alpha) (Gy^−1^) and β (beta) (Gy^−2^) are the radiosensitivity parameters of the LQ model (see also Table 1). Surviving fraction (SF) of tumor cells is the ratio of colonies produced to tumor cells plated, with a correction necessary for plating efficiency. Cell radiosensitivity varies considerably throughout the cell cycle (Steel, 2002; Perez and Brady, 1998). S phase is considered the most radioresistant cell cycle phase (proliferating phase), while all cell cycle phases are more radiosensitive than G0. The model currently uses different values for the radiosensitivity parameters of the LQ model for the S phase (α_s_, β_s_), the remaining proliferating phases G1, G2, M (α_p_, β_p_), and the G0 phase (α_G0_, β_G0_). The values of α_s_, β_s_ and α_G0_, β_G0_ can be derived as perturbations of the (α_p_, β_p_) values (Dionysiou et al., 2006; Carlson et al., 2006):

$$\alpha_{G0}=\alpha_{p}/\text{OER}$$ (2)

$$\beta_{G0=}\beta_{p}/{\text{OER}}^{2}$$ (3)

$$\alpha_{s}=0.6\;\alpha_{p}+0.4\;\alpha_{G0}$$ (4)

$$\beta_{s}=0.6\;\beta_{p}+0.4\;\beta_{G0}$$ (5)

where OER is the Oxygen Enhancement Ratio (Equations 2–5).

When a tumor is radio therapeutically treated, a fraction of cancer cells are lethally hit, i.e., destined to die due to irradiation. Lethally hit cycling tumor cells enter a rudimentary cell cycle that leads to necrotic death after two mitotic divisions. This assumption is based on the experimental finding that most solid cancers activate some cell cycle checkpoints and try to repair the damage. As a result, cells tend to successfully complete one or two mitoses, but, due to accumulating damages, the cells fail to complete more rounds of cell division and succumb in a mitotic catastrophe (Joshi et al., 1982). Marking of a cell as *hit* by the radiation is assumed to take place at the instant of radiation administration.

#### 2.1.2 Model implementation choices—basic notions

The model implementation is based on the consideration of a discrete time and space stochastic cellular automaton, representing the tumor region. More specifically, a three dimensional (3-D) cubic discretizing mesh is superimposed upon the anatomical region of interest. The elementary cube of the mesh is called geometrical cell (GC) The size of the GC can be defined by the user. Typical values considered are 1 × 1 × 1 or 2 × 2 × 2 mm^3^. The exact choice depends on tumor size and available computational resources. Each GC occupied by the tumor corresponds to a cluster of heterogeneous cells found in various states. More specifically, the biological cells residing within each occupied GC are distributed into the five categories mentioned above, i.e., the stem, LIMP, differentiated, apoptotic and necrotic categories. From the mathematical standpoint each cell category defines an equivalence class. Distribution of the cells into the five equivalence classes creates one level of biological cell population partitioning within each GC. At each given instant each stem or LIMP cell can be either proliferating or dormant. Proliferation or dormancy creates another level of cell population partitioning. Cell cycle phases (G1, S, G2, M) introduce a finer partitioning of proliferating cells (stem and LIMP) into subclasses. A further partitioner in the case of therapeutic intervention is treatment hitting, i.e., a Boolean variable denoting whether a biological cell has been hit by treatment. Each occupied GC is assumed to initially contain a fixed *Number of Biological Cells* (NBC). This number is based primarily on radiobiological observations available in pertinent literature, e.g., 10^6^ biological cells/mm^3^ (Begg and Steel, 2002), unless histology based specific data are available. The cytokinetic model regulates the transitions between the various cell states, whereas adequately shaped morphological rules are introduced in order to regulate the cell movement throughout the tumor volume, as described in the following sections.

Time is discretized. Since the duration of the shortest cell cycle phase, which is mitosis (Bast et al., 2000), is approximately 1 h, the discrete time unit, which separates two temporally consecutive virtual scans of the discretizing mesh, is taken equal to 1 h. For any given instant the biological cells belonging to the same cell category and cell cycle phase within a given GC are assumed synchronized. However, biological cells belonging to different GCs or to different categories and cell cycle phases within the same GC are not assumed synchronized. From the computational standpoint a sufficient number of registers are used to describe the state of each GC occupied by the tumor. They include i. a. the number of biological cells residing in each equivalence class and subclass and the time spent at each subclass.

The duration of mitosis phase is considered constant and equal to 1 h (Bast et al., 2000). The rest of the cell cycle phases durations are computed based on (Chu and Sartorelli, 2012) after having taken into consideration the above assumption regarding the constant duration of mitosis. More specifically the following equations are used: T_G1_ = T_S_ = 0.41 (T_c_-T_M_), T_G2_ = 0.18 (T_c_-T_M_), T_M_ = 1 h.

The simulation code has been implemented in C++.

#### 2.1.3 Simulation procedure

**FIGURE 2:**
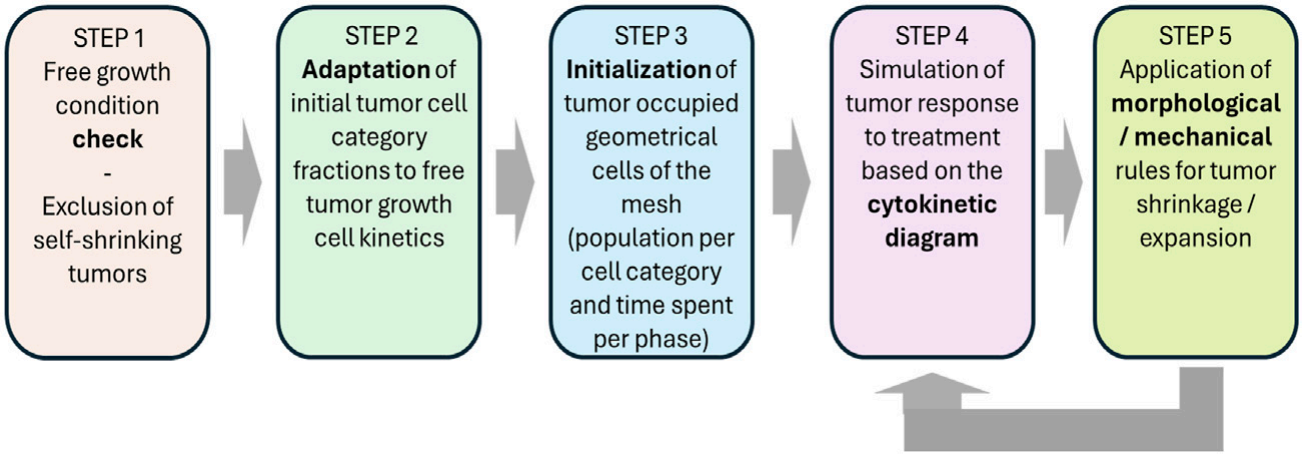
Flow chart of the simulation procedure for a macroscopically homogeneous solid tumor of arbitrary shape.

**FIGURE 3:**
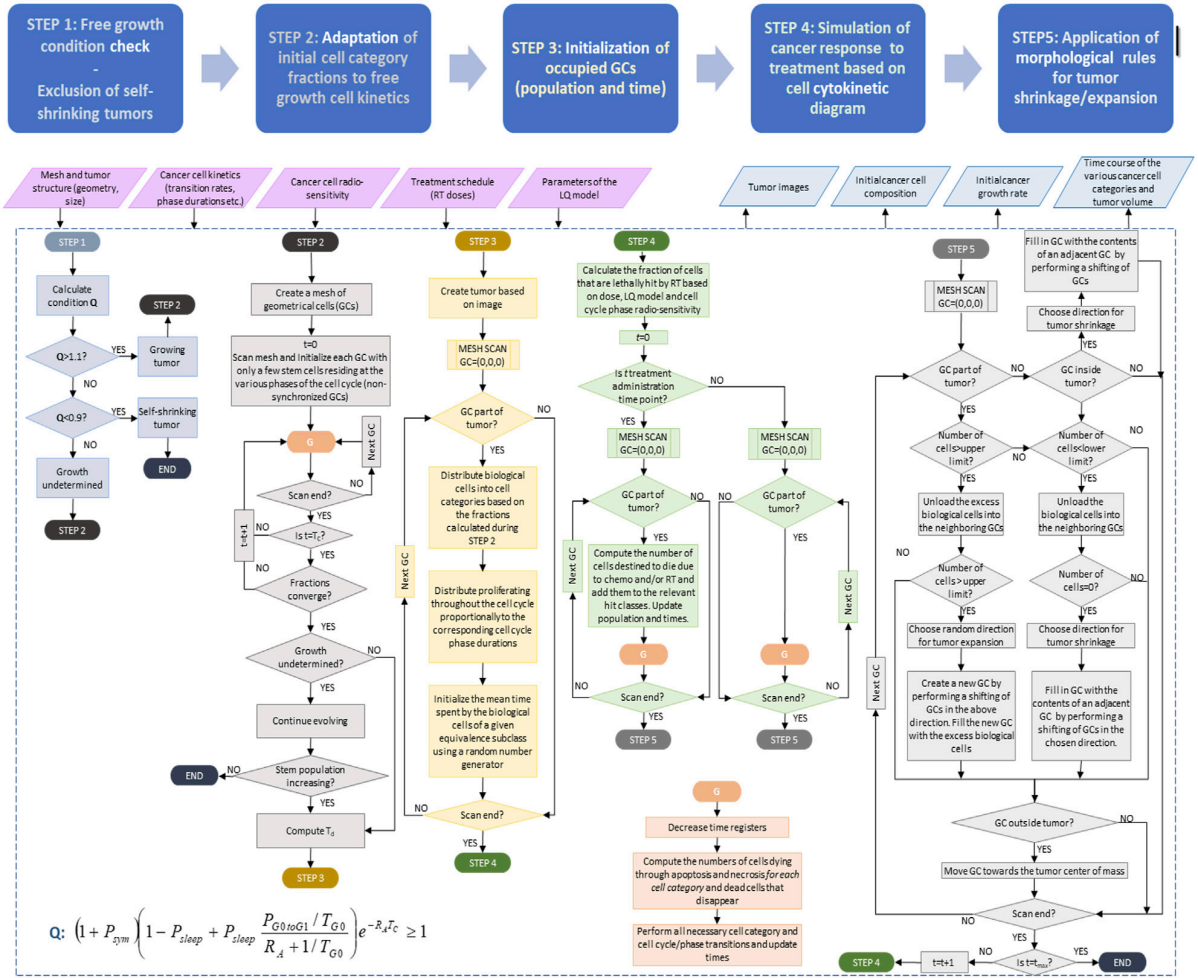
Detailed simulation flow diagram (simulation model description). See also Section 2.1.3 and in this figure. Symbols and abbreviations: RT: radiotherapy, LQ: linear-quadratic model, t: time, GC: geometrical cell of the discretization mesh superimposed onto the anatomic region of interest. *T*_*d*_: doubling time, *P*_*sym*_: fraction of stem cells that perform symmetric division, *P*_*sleep*_: fraction of cells entering the G0 phase following mitosis, *P*_*G0toG1*_: fraction of dormant (stem and LIMP or Limited Mitotic Potential) cells that re-enter cell cycle, *R*_*A*_: apoptosis rate of living stem and LIMP tumor cells (fraction of cells dying through apoptosis per unit time), *T*_*G0*_: G0 (dormant phase) duration, i.e., time interval before a dormant cell enters necrosis, *T*_*c*_: cell cycle duration. Note that the term “chemo” refers to the eventual (additional) administration of chemotherapy in a more generic context. which is not., however, addressed in this paper.

A flowchart of the simulation algorithm is depicted in Figure 2, whereas a more detailed description is provided in Figure 3. The major steps involved are described below.

##### 2.1.3.1 STEP 0: tumor definition

The model supports the simulation of three-dimensional tumors of arbitrary shape, as well as the division of tumor area into regions of different metabolic activity (e.g., necrotic and proliferative). Mesh initialization involves the definition of occupied and non occupied GCs, based on the available patient-specific imaging data. Occupied GCs (i.e., GCs that belong to the tumor region) can be further subdivided into necrotic or proliferative, provided that the relevant info has been foreseen in the segmented tumor images. The values assigned to the model input parameters can be defined for each type of GC in relation to the considered metabolic subregions. Patient-specific data such as histological data (e.g., type, stage and differentiation grade) and molecular data, as well as other tumor specific data, can be incorporated for further refinement of the values attributed to the model input parameters. For the two prostate tumor cases considered, the Gleason score is ≤8. Therefore, the fact that we are dealing with prostate cancer has dictated boundaries for tumor growth fraction and doubling time, whereas the fact that Gleason score is ≤8 has dictated a practical absence of necrosis. Apart from this type of histological data, the specific examples presented exploit MRI data pertaining to the spatial boundaries and the volume of the tumor before and after treatment (through tumor 3D reconstruction from the available 2D MRI segmented via slice interpolation slices) and the precise radiotheraputic treatment scheme for each patient. More generally, values of critical proliferation features of the virtual tumors, e.g., growth rate, growth fraction, the fraction of stem cells and necrotic and apoptotic cells, are informed by clinical studies in literature for prostate cancer. For example, the literature review conducted reveals a very wide range of tumor doubling times (based on PSA measurements) spanning from a couple of months to infinity (stable tumors), while growth fraction is usually low. Furthermore, necrosis is not seen in prostate tumors characterized by the Gleason scores of the cases considered in our study (≤8). The combination of parameter values ensures that the percent of necrosis, is negligible in all virtual tumor implementations, in line with literature observations. More information on the parametrization methodology along with the mathematical derivations used to link proliferation features with model parameters can be found in (Kolokotroni et al., 2016; Kolokotroni et al., 2024; Kyroudis et al., 2019). In case of macroscopically homogeneous tumors, all occupied GCs are characterized by identical values. It should be noted that in the absence of 3D imaging data the tumor shape is approximated with a triaxial ellipsoid. The length of each axis of the ellipsoid is assumed equal to the maximum tumor diameter(s) as measured based on, e.g., ultrasound examination.

##### 2.1.3.2 STEP 1: free growth condition check

Based on the cytokinetic model described previously, stem cells are responsible for sustaining the cancer and their behavior plays a determinant role for cancer free growth evolution. Depending on the values assigned to the model parameters that describe the life course of stem cells, it is possible to simulate cancer with variable degree of aggressiveness in terms of growth rate. Furthermore, there exist certain “forbidden” value combinations of these parameters that lead to biologically irrelevant cancers, i.e., cancers that diminish over time of their own accord, unable to sustain growth.

A condition is applied to check whether the value combination of input parameters leads to a growing or self-diminishing cancer. The condition has been derived (Kolokotroni et al., 2011) from an analytical treatment of model assumptions following the methodology of (Bertuzzi et al., 1997) (Equation 6).

$$\left(1+P_{\text{sym}}\right)\;\left(1-P_{\text{sleep}}+P_{\text{sleep}}\frac{P_{G0\text{toG}1}/T_{G0}}{R_{A}+1/T_{G0}}\right)\;e^{-R_{A}T_{C}}\geq 1$$ (6)

In order to take into account any divergence between the simulation results and the above condition we assume that for values of the left side of the above inequality lower than 0.9 cancer free growth cannot be sustained, whereas for values above 1.1 free growth is ensured. In the middle value range the cancer free growth is checked based on simulation results during the turmogenesis process.

##### 2.1.3.3 STEP 2: adaptation of initial tumor cell category fractions to free tumor growth cell kinetics

The technique applied for the determination of tumor’s cell composition is critical, so as to avoid latent artificial tumor growth behaviors. A decrease in tumor volume followed by a volume increase is a very common pattern (Kolokotroni et al., 2008). In order to avoid an abnormal free growth behavior at the beginning of the simulation, the automatic tumor initialization methodology has been developed (Stamatakos et al., 2010). The principle of the tumor constitution initialization technique is to start with a small number of stem cells and with specific cell category transition rates that are assumed to hold true for a relatively small time interval around the treatment baseline. Specific values are assigned to the phase durations and transition rates. Gradually, all cell categories and phases become populated and after sufficient time the relative cell categories populations tend to reach an equilibrium state. If in subsequent simulations the GCs are initialized using the cell category/phase relative population values corresponding to this equilibrium state, a mathematically monotonous and biologically realistic free growth behavior will be achieved. The challenge is to successfully locate the point beyond which equilibrium has been achieved and use the relative populations (or ‘‘fractions of populations’’) after that point for the correct initialization of the tumor. Certain combinations of category/phase transition rates cannot sustain tumor growth (see Section 2.1.3.2 for the condition for monotonic free growth). In such cases the method will correctly fail to create the initial tumor and a relevant warning message will be issued by the simulation system. More specifically, the technique consists of the following steps: i) A limited number of geometrical cells NGCs are considered. ii) Each GC initially contains a small number of stem cells, e.g., 100, residing in the various cell cycle phases (G1, S, G2, M) and the G0 phase. iii) *Time initialization*, i.e., the time already spent by clustered stem cells in the phase they reside is assigned using a pseudorandom generator. iv) Different random number sets are assigned to different GCs. The aim is to avoid artificial synchronizations which would result in the group of GCs considered behaving as one big GC. v) The system is left to evolve and produce all cell category populations (distributed to the various cell phases). vi)The code execution has to continue until equilibrium is reached and the various cell categories population percentages have been stabilized. The parameter value ranges appearing in Section 3.1.1 in conjunction with further literature information pertaining to the prostate tumor subtypes considered can be exploited by this procedure in the case of prostate cancer.

##### 2.1.3.4 STEP 3: initialization of tumor occupied geometrical cells of the mesh

The biological cells residing within each geometrical cell of the mesh are distributed into the 5 cell categories (i.e., stem, LIMP, differentiated, apoptotic, necrotic), based on the fractions calculated during the previous step. The initial distribution of the proliferating cells throughout the cell cycle phases (G1, S, G2, M) is assumed to be proportional to the corresponding cell cycle phases durations. The mean time spent by the biological cells of a given equivalence subclass in the same subclass is initialized using a random number generator (Monte Carlo technique). The time under consideration can vary between 0 and the maximum time of the corresponding phase. As mentioned above biological cells belonging to different GCs or to different categories and cell cycle phases within the same GC are not assumed synchronized.

At each hourly time step, the discretizing mesh covering the anatomical region of interest is virtually scanned in order to apply the basic rules that govern the spatiotemporal evolution of the tumor system. For practical reasons each complete virtual scan can be viewed as consisting of two mesh scans: one dealing with the application of the metabolic, cytokinetic, radiobiological laws and rules and one dealing with the mechanical rules.

##### 2.1.3.5 STEP 4: simulation of tumor response to treatment based of the cytokinetic diagram (first mesh scan)

The first scan aims at updating the state of each GC according to the proposed and adopted approximate cytokinetic model of Figure 1. The time registers of the various cell subclasses within each geometrical cell are updated and the cytokinetic diagram is applied within each GC as follows. Spontaneous apoptosis induced cell loss from each non treatment perturbed cell cycle phase and the G0 phase is calculated for each cell category based on the spontaneous apoptotic rates assumed. Any necessary transitions between equivalence subclasses (G1→S, S→G2, G2→M, M→G1 or M→G0) take place for biological cells clustered in the same subclass. The latter depends on the updated value of the corresponding time registers. If the mean time that the clustered cells have spent in the corresponding phase has become equal to or larger than the phase duration then the cells enter a new phase and equivalence subclass.

In any one of the cases of dormant (including stem and LIMP), differentiated, necrotic and apoptotic cells a fraction of the corresponding subclass (es) population may be transferred to another subclass or disappear from the tumor at each time step according to the cytokinetic model (Figure 1). Therefore the following transitions may take place. For stem and LIMP cells: G0→G1 or G0→Necrosis or G0→Apoptosis. For differentiated cells: Differentiated→Necrosis or Differentiated→Apoptosis. For dead cells of any mitotic potential category: Apoptosis→Cell disappearance, Necrosis→Cell disappearance. Most of the corresponding rates are parameters of the model (Table 1).

Cell killing by irradiation is described by the Linear-Quadratic (LQ) Model (1).

##### 2.1.3.6 STEP 5: application of morphological/mechanical rules for tumor shrinkage/expansion (second mesh scan)

The second scan aims at simulating tumor expansion or shrinkage, while preserving a roughly uniform cell density throughout the tumor volume. To this end, adequately shaped morphological rules are introduced, which may lead to tumor expansion, as is the case in free tumor growth, or no change in tumor volume or tumor shrinkage as is usually the case after treatment administration. The adopted morphological rules (Stamatakos et al., 2006) aim at preserving the cohesion and initial shape figure of the tumor under the assumption that the mechanical properties of the surrounding normal tissues are invariant throughout the simulation, while preventing the tumor to acquire an artificial shape. During the above process artificial tumor fragmentation may, however, occur and a special procedure has been devised in order to achieve macroscopic tumor cohesion (Stamatakos et al., 2006). The latter usually characterizes tumor shrinkage following treatment.

For practical reasons at any given time point the total cell population that can be accommodated in each GC is allowed to fluctuate between a minimum (0.9*NBC) and a maximum (1.1*NBC) value. If the total population exceeds the maximum value of 1.1*NBC then a procedure is initiated that attempts to unload the total GC population minus NBC to neighboring GCs (26 GC neighborhood is considered) possessing empty space, i.e., GCs with total cell population less than NBC. The procedure starts from the neighboring GC possessing the maximum free space. If two or more neighboring GCs possess the same free space then a random number generator is used so as to select the visiting order of the GCs. The procedure is repeated until all the excess cells have been transferred, provided this is possible. If the procedure fails to reduce the total population of the GC under consideration below the upper limit (maximum value) then an adjacent GC is freed from its contents which are moved outwards. The latter push the contents of a chain of geometrical cells outwards too. The excess contents of the GC under consideration are placed into the newly freed adjacent GC. The previous process leads to differential tumor expansion. The position of the GC to be freed from its contents relative to the GC with the excess contents is determined using a random number generator. The shifting of the chain of GCs mentioned above can take place along any randomly selected direction. The direction is selected based on a random number generator.

On the other hand if the GC’s total cell population is below the minimum value then a similar procedure attempts to unload all cells to neighboring GCs possessing free space. If the GC becomes empty then a chain of GC contents is shifted towards the GC under consideration so as to fill the vacuum generated. The latter leads to differential tumor shrinkage. Shifting of the GC content chain takes place as follows. Six lines of random direction are chosen based on a random number generator. The outermost non-empty GC along each one of these directions is detected and its “6-Neighbor” GCs belonging to the Tumor (NGCT) are counted. The direction corresponding to the maximum NGCT is selected.

The above procedure, however, may give rise to the following “side effects”. (a) Tumor fragmentation: some GCs belonging to the tumor become separated from the main tumor mass. (b) Vacuum enclosures: holes that correspond to empty GCs are created inside the tumor. In order to avoid the occurrence of the above side effects an algorithm has been developed that (I) detects tumor occupied GCs that are surrounded by empty GCs in a “6-GCs Neighborhood” and moves their contents (by 1 GC at each time step) towards the tumor’s center of mass. The direction of movement is chosen based on the minimum distance of the GC under consideration from the center of mass along the x, y, z coordinates. The corresponding quantity to be calculated each time is the following:

$$\min\;\left\{\text{abs}\left(\text{GC}.x-\text{center}.x\right),\text{abs}\left(\text{GC}.y-\text{center}.y\right),\text{abs}\left(\text{GC}.z-\text{center}.z\right)\right\}$$ (7)

where abs () denotes absolute value, GC. x, GC.y and GC. z are the x, y and z coordinates of the GC respectively and center. x, center. y and center. z are the x, y and z coordinates of the tumor’s center of mass respectively (Equation 7). If more than one direction is characterized by the same minimum distance then a random number generator is used for the selection of the movement direction. (II) Detects empty GCs that are surrounded by occupied GCs in a “6-GCs Neighborhood” and fills them with the contents of adjacent GCs by applying the tumor shrinkage procedure described above.

## 3 Results

### 3.1 Preliminary parametric studies: qualitative assessment

Following technical verification of the proposed model, an indicative sensitivity analysis and a preliminary adaptation study are presented in this section. Since our adaptation approach can best demonstrate the histological handling strategy of the proposed model, it is described first, i.e., before the sensitivity analysis, where histological handling is of great importance. Both investigations also provide an initial confirmation of the correct operation of the core simulation model of the OncoSimulator digital twin.

#### 3.1.1 A preliminary adaptation study

**FIGURE 4:**
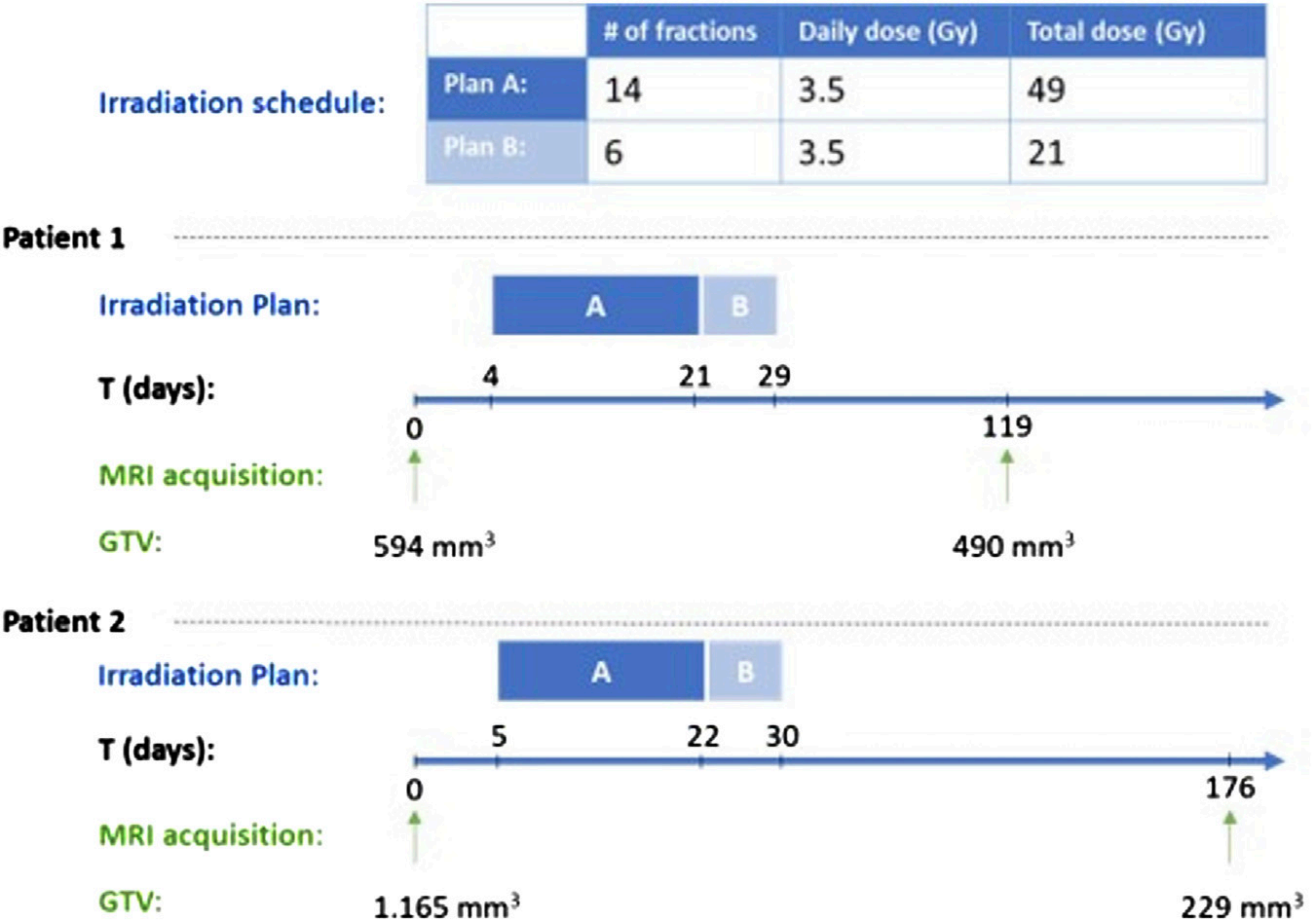
Radiation schedule and tumor measurement time points for the two clinical cases considered. GTV: Gross Tumor Volume.

In this section a proof of concept clinical adaptation paradigm is presented. More specifically, the core of the In Silico Oncology and In Silico Medicine Group discrete model presented previously has been applied to the case of prostate cancer neoadjuvant radiotherapy treatment. More specifically, the model has been applied to a clinical data set of two patients with primary prostate cancer, treated with two consecutive plans, A and B (Figure 4). Radiation is administered daily with a pause during weekend. The patient specific data that have been exploited by the model are the applied radiotherapeutic scheme (fraction dose, administration instants) and the volume reduction as defined from the 3D image of the tumor as reconstructed from MRI imaging data (Figure 4). The sets of imaging data were provided for two time instants before and after the completion of the treatment. Due to the non availability of data related to any distinct internal metabolic regions, the virtual tumors implemented are homogeneous. The study is focused on quantifying the radiosensitivity of the specific tumor, i.e., the parameters α (Gy^-1^) and β (Gy^-2^) of the LQ model. It is noted that α and β refer to the most radiosensitive proliferating phases, i.e., G1, G2 and M. Here, a plausible value range of parameter α is suggested following the exploitation of cancer-specific literature data, the actual clinical data and the simulation outcome.

**FIGURE 5:**
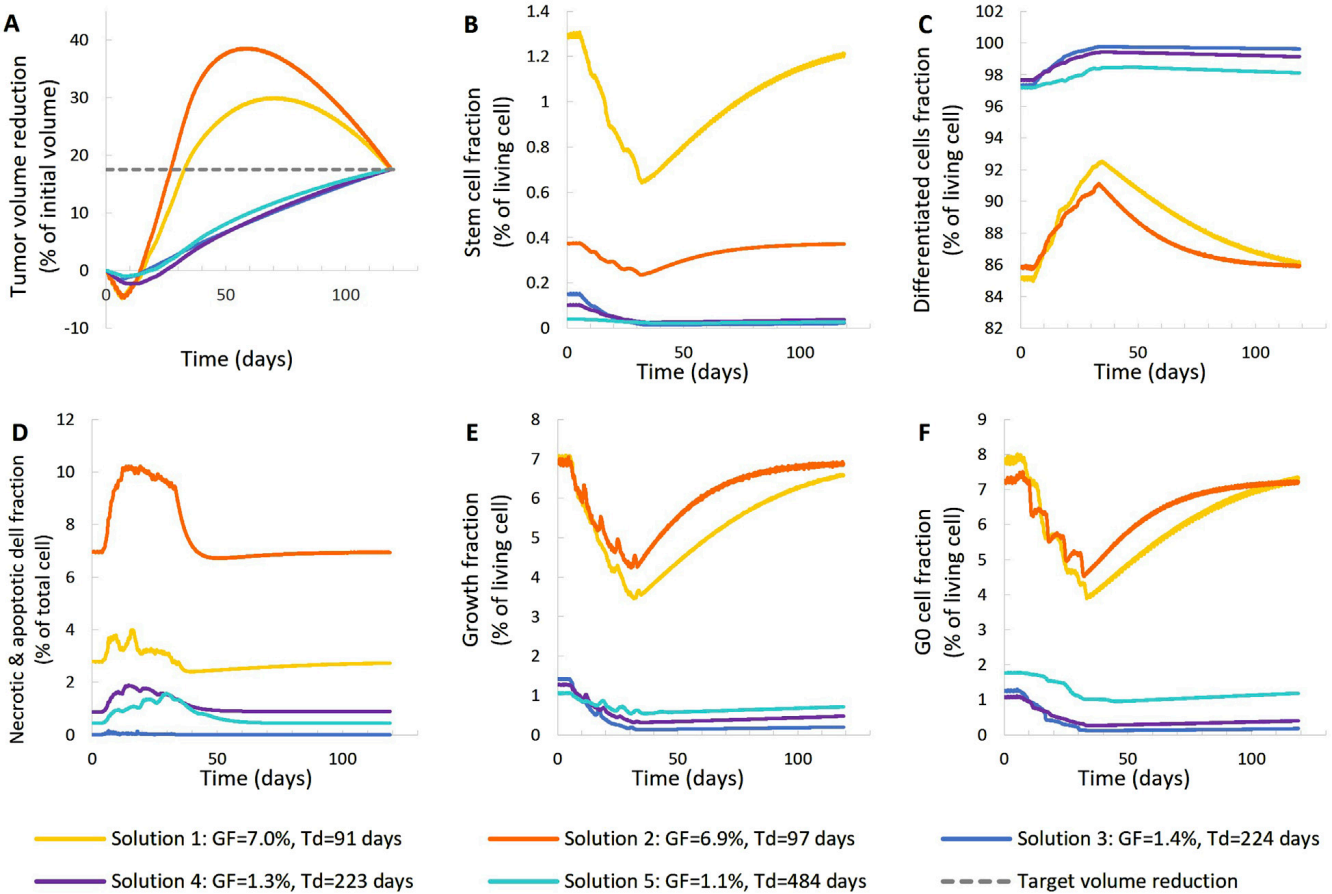
Comparison of five different virtual tumors (Table 2), all compatible with patient’s volumetric data (solutions) (Figure 4, Patient 1), in terms of the time evolution of tumor volume reduction **(A)** and the fraction of selected tumor subpopulations [stem cells **(B)**, terminally differentiated cells **(C)**, cells that have died through necrosis or apoptosis **(D)**, proliferating cells in the active cell cycle **(E)** and cells in a reversible G0 state **(F)**]. The radiation schedule of Figure 4 (Patient 1) has been simulated. Symbols and abbreviations: GF: growth fraction, T_d_: tumor doubling time.

**FIGURE 6:**
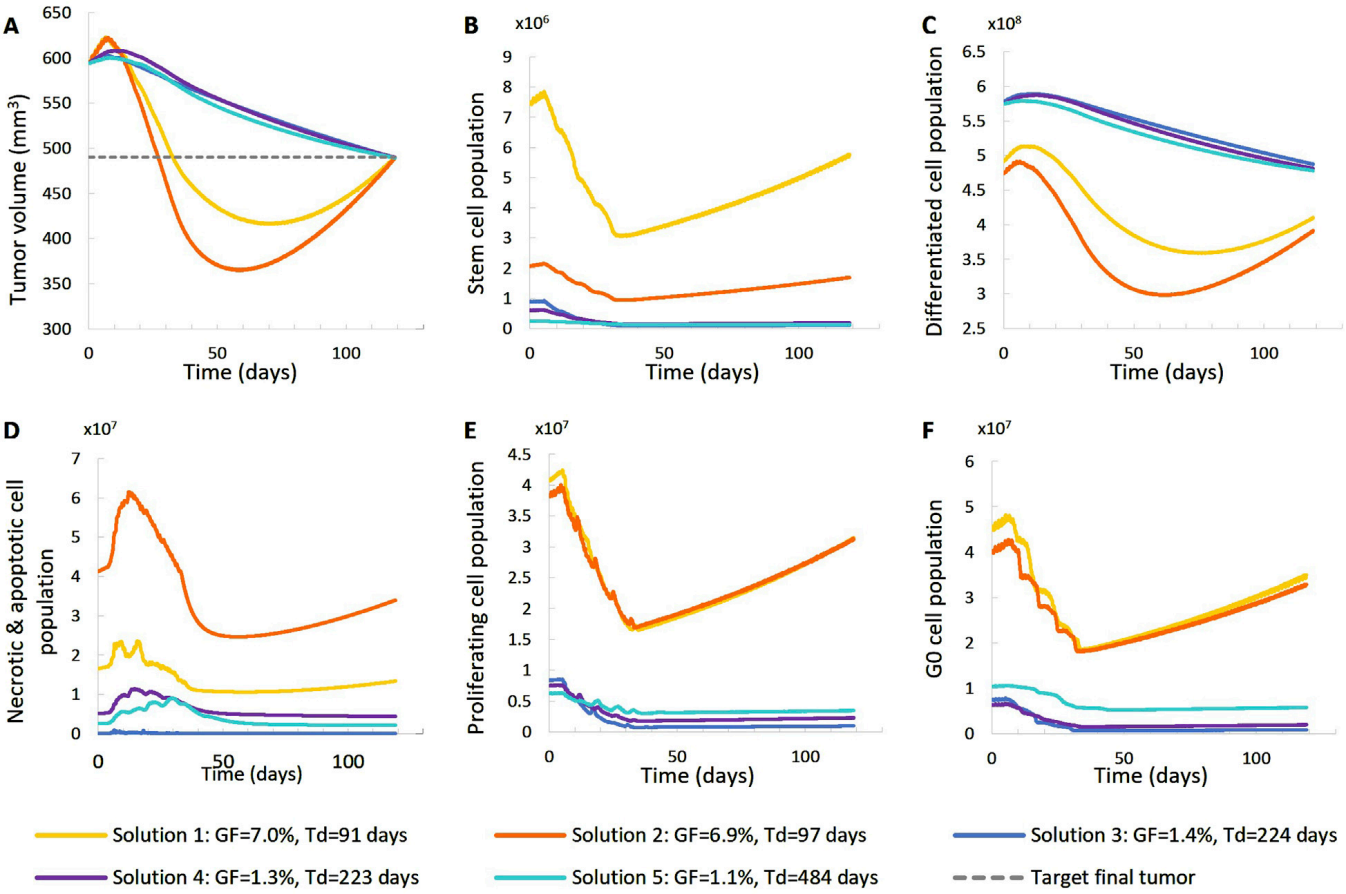
Comparison of five different virtual tumors (Table 2), all compatible with patient’s volumetric data (solutions) (Figure 4, Patient 1), in terms of the time evolution of tumor volume **(A)** and selected tumor subpopulations [stem cells **(B)**, terminally differentiated cells **(C)**, cells that have died through necrosis or apoptosis **(D)**, proliferating cells in the active cell cycle **(E)** and cells in a reversible G0 state **(F)**]. The radiation schedule of Figure 4 (Patient 1) has been simulated. Symbols and abbreviations: GF: growth fraction, Td: tumor doubling time.

In the following, the results of the clinical adaptation are presented. We examined five different, biologically reasonable, *in silico* representations (virtual tumors) of the patient. For each of these five virtual tumors, we adjusted the α, β parameters of the LQ model so that the observed volume reduction of the tumor is achieved (i.e. 17.51%). Table 2 lists the parameter values of the five solutions. Table 3 summarizes the growth rate and the resulting tumor cell composition at the start of the simulation for each solution. Figures 5, 6 include graphs expressing the time evolution of various tumor characteristics.

**TABLE 2:** Parameter values for 5 virtual tumors created.

Parameter	Solution-1	Solution-2	Solution-3	Solution-4	Solution-5
T_c_ (h)	31	30	31	30	31
T_G0_ (h)	131	113	146	109	373
T_N_ (h)	29	98	5	143	186
T_A_ (h)	7	16	1	4	17
N_LIMP_	8	7	9	7	7
R_A_ (10^−4^ h^−1^)	44.999	5.425	69.285	46.744	10.230
R_NDiff_ (10^−4^ h^−1^)	5.951	2.775	0.189	0.600	0
R_ADiff_ (10^−4^ h^−1^)	4.062	16.102	0.845	1.102	2.591
P_G0toG1_ (h^−1^)	0.5	0.5	1	1	1
P_sleep_	0.308	0.255	0.288	0.289	0.179
P_sym_	0.474	0.190	0.453	0.278	0.090
a/β (Gy)	3	3	3	3	3
OER	1.960	2.187	2.989	2.942	2.388
α (10^−3^ Gy^−1^) of patient 1	10.068	9.969	25.998	16.496	9.336
α (10^−3^ Gy^−1^) of patient 2	26.795	28.185	—	—	—

No solutions 3-5 of α in the value range 0.0-0.4 (Gy^-1^) could be found for Patient 2 (see Section 3.1.1.2).

**TABLE 3:** Characteristics of 5 virtual tumors generated.

	Doubling time (days)	Growth rate (10^−4^ h^−1^)	Necrotic and apoptotic cell fraction (%)	Growth fraction (%)	Stem cell fraction (%)	Differentiated cell fraction (%)	G0 cell fraction (%)
Solution-1	91	3.189	2.785	7.046	1.291	85.145	7.809
Solution-2	97	2.986	6.954	6.911	0.374	85.835	7.254
Solution-3	224	1.291	0.007	1.411	0.150	97.337	1.252
Solution-4	223	1.295	0.875	1.273	0.102	97.651	1.075
Solution-5	484	0.597	0.447	1.057	0.040	97.176	1.768

In order to best adjust the model to the available prostate cancer radiotherapy literature data, a literature review has been conducted in order to identify the approximate value ranges of the following major aspects of the model. It is noted, however, that due to remarkable differences in the value ranges reported in literature, value ranges larger than the ones already identified in literarure have been considered in the present analysis.

**Cell cycle duration** (e.g., Cunningham and You, 2015; Wang et al., 2019; Liu et al., 2020) [cell cycle duration T_c_: ∼30 h], **quiescence** (e.g., Pulianmackal et al., 2021) [G0 (dormant phase) duration T_G0_: 4–20 days extension of (Durand and Sham, 1998)], **growth rate** (e.g., Lee et al., 1995; Howard et al., 2017; Ng et al., 2009) [doubling time Td: ∼100, 200 and 500 days ], **growth fraction** (e.g., Khor et al., 2009; Wilkins et al., 2018; Richardsen et al., 2017) [growth fraction GF:<10%], **spontaneous apoptosis** (e.g., Dachille et al., 2008) [apoptotic index = percentage of apoptotic cells in a tumor cell population: ∼0.5%], **hypoxia and necrosis** (e.g., McKeown, 2014; Hompland et al., 2018; Bhattacharya et al., 2019; Mai et al., 2019) [prostate cancer is hypoxic tolerant, no necrosis for tumors of Gleason Score ≤8,], **cancer stem cells** (e.g., Franco et al., 2016; Ishizawa et al., 2010; Li et al, 2020; Skvortsov et al., 2018) [percentage of cancer stem cells: <∼1%)], **radiosensitivity** (e.g., Carlson et al., 2004; van Leeuwen et al., 2018; Wang et al., 2003; Dasu and Toma-Dasu, 2012) [alpha parameter of the LQ model: 0.026 Gy^-1^ – 0.34 Gy^-1^; α/β: 3 Gy, Five virtual tumors with specific proliferation and radiosensitivity characteristics were considered. Initially, random values within the specified ranges were assigned to model parameters related to free growth: *T_C_*, *T_G0_*, *R_A_*, *P_G0toG1_*, Psym, NLIMP, and radiosensitivity: α/β and OER were considered. For each combination of these parameter values, the parameters *P_sleep_* and *R_ADiff_*, were adjusted to achieve the specified tumor doubling time (Td) and growth fraction (GF) as per (Kolokotroni et al., 2011; Kolokotroni et al., 2016). Finally, the radiosensitivity parameter alpha (α) of the Linear-Quadratic (LQ) model was adjusted to match the observed tumor size reduction. This adaptation of α was carried out automatically using an optimization procedure. Specifically, the fzero command in MATLAB was employed to find the root of the difference between the observed volume reduction (based on the two DICOM data sets) and the volume reduction of the simulated tumor.

##### 3.1.1.1 First patient–virtual tumors

The following observations can be made (Figures 5, 6). The five virtual tumors refer to three different proliferation scenarios in terms of doubling time and growth fraction. In the virtual tumors with a short doubling time (less than or comparable to the observation window) (Solution-1 and Solution-2) two phases can be distinguished: the regression phase and the regrowth phase. In contrast, in the virtual tumors with a longer doubling time, only one phase is distinguished, the regression one, and the volume decrease is monotonous until the time of the follow-up image acquisition. The radiosensitivity parameter α (Gy^-1^) ranges between 0.01 and 0.024 for all virtual tumors, implying a radioresistant tumor.

A good fitting between the simulation results and the patient volumetric data has been achieved in all cases. The respective deviation has been less than 0.1%. The results indicate a feasible value range of the radiosensitivity parameter α (Gy^-1^). However, a larger number of possible virtual tumor implementations might further support the generality of the approach presented. This will be the subject of future work.

##### 3.1.1.2 Second patient–virtual tumors

**FIGURE 7:**
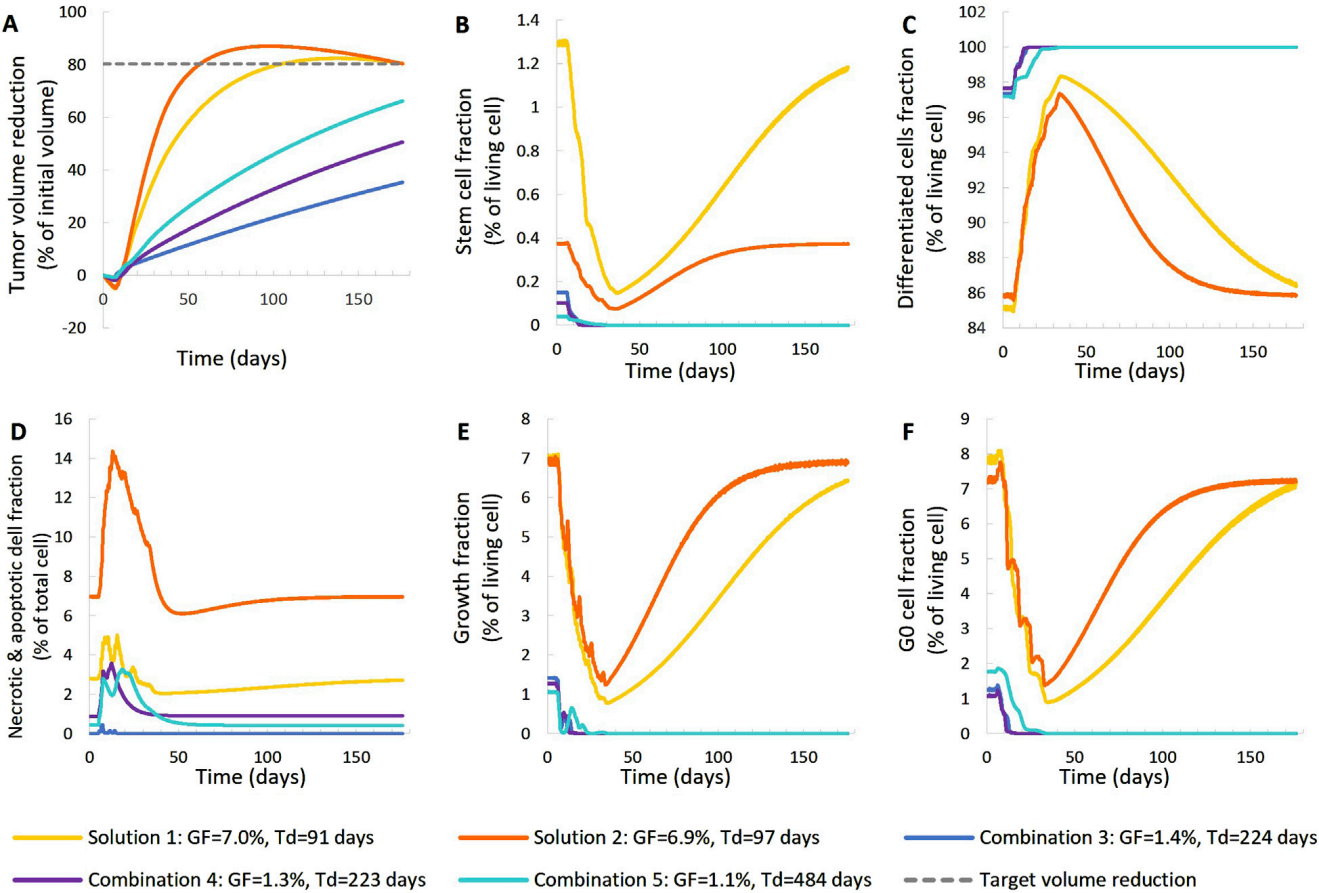
Comparison of five different virtual tumors (Table 2), all compatible with patient’s volumetric data (solutions) (Figure 4, Patient 2), in terms of the time evolution of tumor volume reduction **(A)** and the fraction of selected tumor subpopulations [stem cells **(B)**, terminally differentiated cells **(C)**, cells that have died through necrosis or apoptosis **(D)**, proliferating cells in the active cell cycle **(E)** and cells in a reversible G0 state **(F)**]. The radiation schedule of Figure 4 (Patient 2) has been simulated. Symbols and abbreviations: GF: growth fraction, Td: tumor doubling time.

**FIGURE 8:**
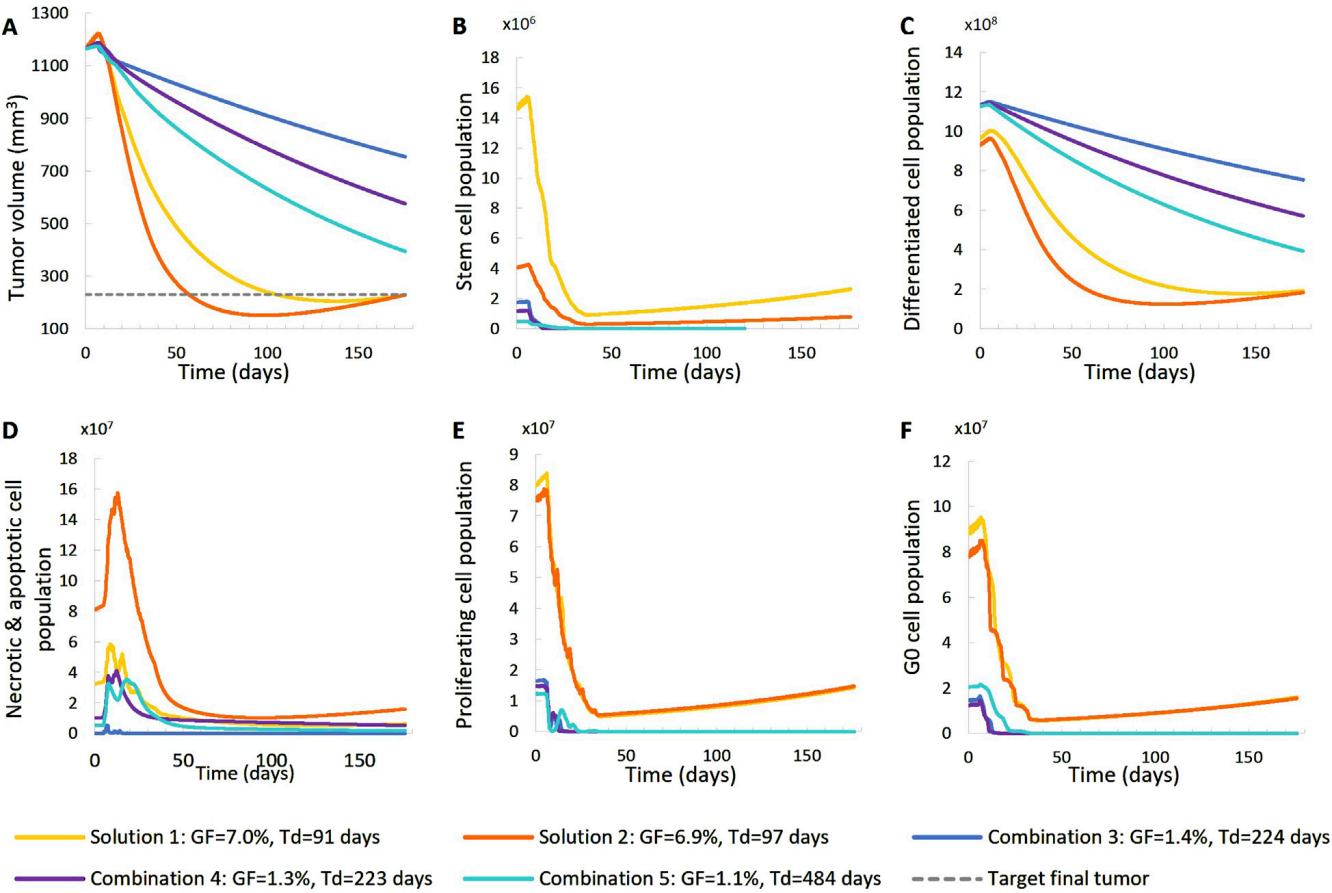
Comparison of five different virtual tumors (Table 2), all compatible with patient’s volumetric data (solutions) (Figure 4, Patient 2), in terms of the time evolution of tumor volume **(A)** and selected tumor subpopulations [stem cells **(B)**, terminally differentiated cells **(C)**, cells that have died through necrosis or apoptosis **(D)**, proliferating cells in the active cell cycle **(E)** and cells in a reversible G0 state **(F)**]. The radiation schedule of Figure 4 (Patient 2) has been simulated. Symbols and abbreviations: GF: growth fraction, Td: tumor doubling time.

For patient 2 (Figures 7, 8), only the high proliferation profile is compatible with the observed tumor regression. Virtual tumors with a very low growth fraction (∼1%) (combinations 3-5 in Figures 7, 8) could not regress to the observed magnitude, even with high radiosensitivity (α = 0.4 Gy^−1^). The radiosensitivity parameter α (Gy^−1^) ranges between 0.01 and 0.024 for patient 1 and is approximately 0.03 for patient 2 (for the virtual tumors of Solutions 1-2 implemented), indicating higher radiosensitivity. The radiosensitivity parameter for Combinations 3-5 (Patient 2) has been taken α = 0.4 Gy^−1^. These estimated radiosensitivities correlate with the observed volume reductions, with patient 1 showing only an ∼18% reduction, while patient 2 exhibits an ∼80% reduction.

##### 3.1.1.3 Three dimensional visualization of model predictions

**FIGURE 9:**
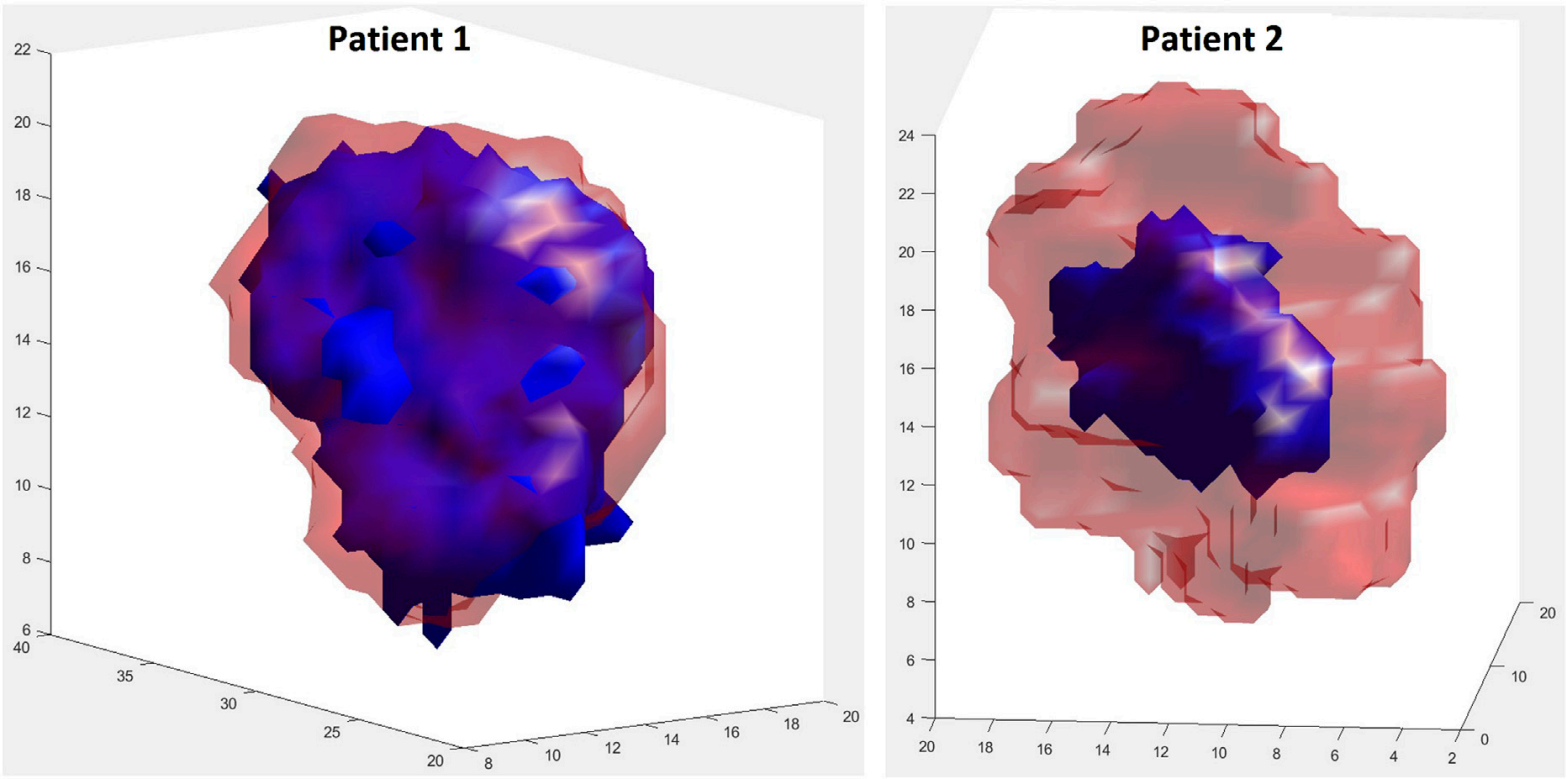
Visual comparison of shape between initial true (red, transparent) and final simulated (blue) tumors at the respective first and second imaging time points. For patient 1, during the simulation period (day 1–119), the tumor volume decreases by 17.51%. For patient 2, during the simulation period (day 1–176), the tumor volume decreases by 80.34%. Solution 1 of Table 2 has been simulated for both patients.

Since the specific tumors from two patients considered appeared microscopically homogeneous, only their initial shapes and their initial volumes were extracted from the corresponding MRI imaging data and utilized. Figure 9 provides the initial shapes and volumes of the two tumors considered along with their simulated shrinked shapes and volumes. It is noted, however, that the proposed model can handle spatial inhomogeneities of parameters such as oxygenation. The final shrinked tumor predicted shapes are relatively close to their actual MRI reconstructed counterparts, based on rather qualitative visual inspection.

#### 3.1.2 Parametric and sensitivity analysis

##### 3.1.2.1 Parametric analysis

Here, we present a qualitative assessment of key model parameters, as a preliminary sensitivity analysis study. Patient 1 has been considered as reference. (Table 2). We present 6 variations of a baseline solution (Case-0 that corresponds to Solution-5 of the preliminary adaptation study), which corresponds to a value assignment to the model parameters that accurately represents the patient considered *in silico*. In each variation, we tweak the values of a limited number of model parameters, in order to observe the isolated effect that each parameter has on the virtual patient scenario. That way, we create 6 different exploratory cases of the baseline.

**FIGURE 10:**
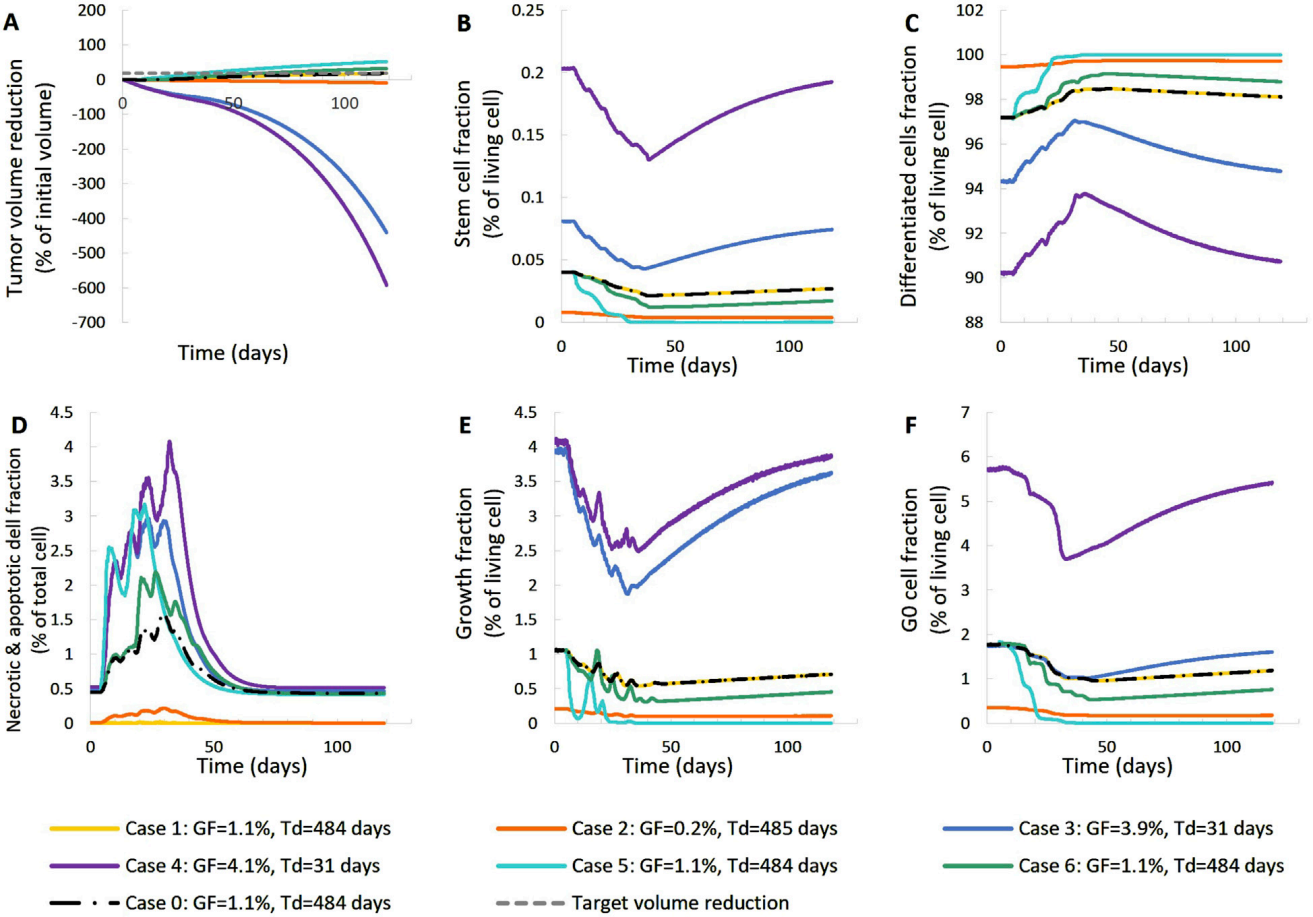
Effect of model input parameters on the time evolution of tumor volume reduction **(A)** and the fraction of selected tumor subpopulations [stem cells **(B)**, terminally differentiated cells **(C)**, cells that have died through necrosis or apoptosis **(D)**, proliferating cells in the active cell cycle **(E)** and cells in a reversible G0 state **(F)**]. The radiation schedule of Figure 4 (Patient 1) has been simulated. Case-0 is a solution of the clinical case considered, i.e., it is compatible with the patient’s volumetric data (Figure 4, Patient 1). Case-1 up to Case-6 have been derived from Case-0 after changing the value of a limited number of model parameters (Table 4). Case-1 and Case-2 correspond to scenarios of small dead cell compartment, Case-3 and Case-4 correspond to more proliferative profiles and Case-5 and Case-6 simulate tumors of higher radiosensitivity. Symbols and abbreviations: GF: growth fraction, T_d_: tumor doubling time.

**FIGURE 11:**
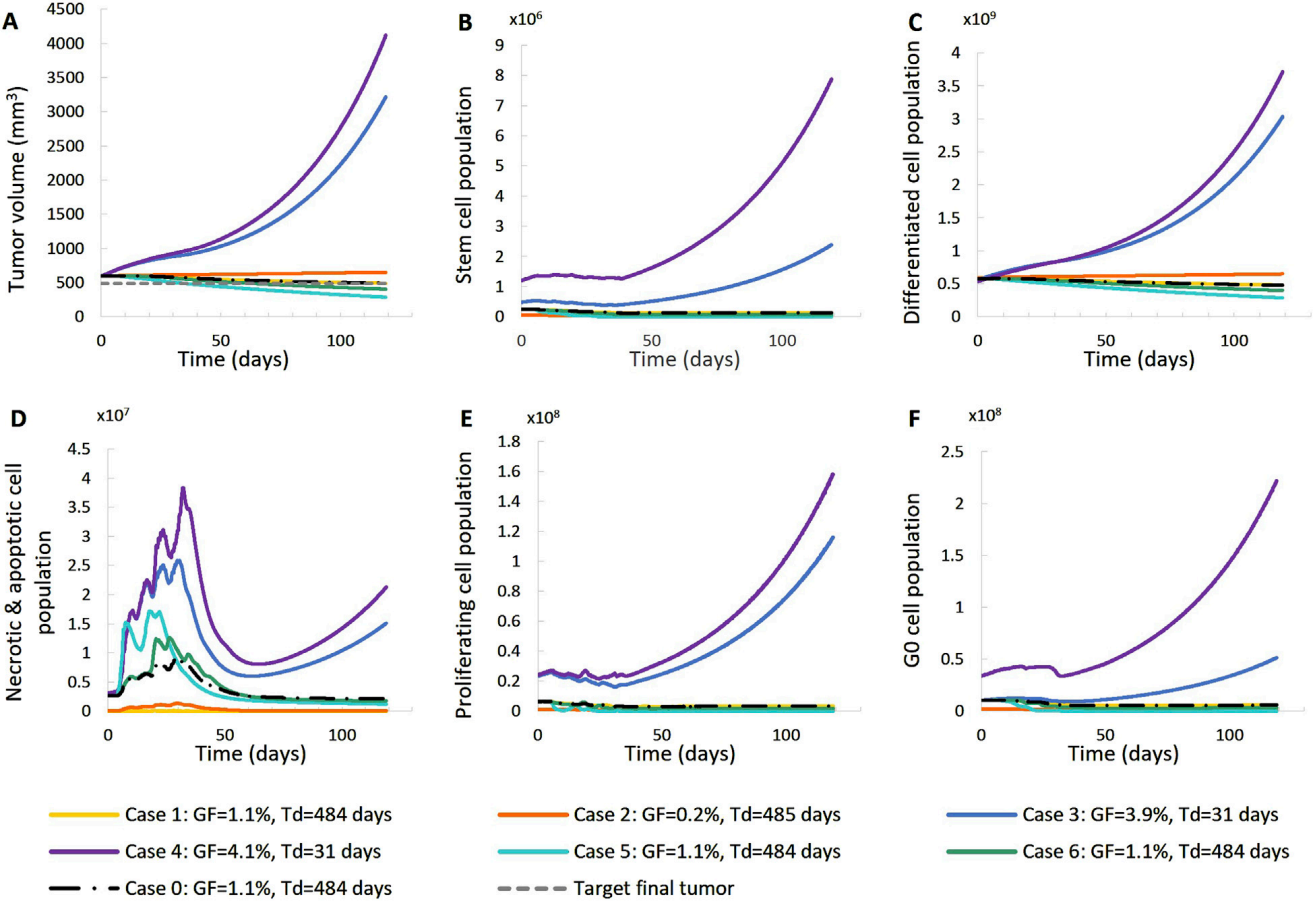
Effect of model input parameters on the time evolution of tumor volume **(A)** and selected tumor subpopulations [stem cells **(B)**, terminally differentiated cells **(C)**, cells that have died through necrosis or apoptosis **(D)**, proliferating cells in the active cell cycle **(E)** and cells in a reversible G0 state **(F)**]. The radiation schedule of Figure 4 (Patient 1) has been simulated. Case-0 is a solution of the clinical case considered, i.e., it is compatible with the patient’s volumetric data (Figure 4. Patient 1). Case-1 up to Case-6 have been derived from Case-0 after changing the value of a limited number of model parameters (Table 4). Case-1 and Case-2 correspond to scenarios of small dead cell compartments, Case-3 and Case-4 correspond to more proliferative profiles and Case-5 and Case-6 simulate tumors of higher radiosensitivity. Symbols and abbreviations: GF: growth fraction, T_d_: tumor doubling time.

Table 4 lists the parameter values of the six different cases (seven together with the baseline Case-0). Table 5 summarizes the growth rate and the resulting tumor cell composition at the start of the simulation for each case, while Figures 10, 11 include graphs expressing the time evolution of the relevant characteristics.

**TABLE 4:** Parameter values for the 6 different cases (7 including the baseline Case-0) tested with the imaging data of the patient considered for the qualitative study of the OncoSimulator. Patient 1 has been considered as reference.

Parameter	Case-0	Case-1	Case-2	Case-3	Case-4	Case-5	Case-6
T_c_ (h)	31	31	31	31	31	31	31
T_G0_ (h)	373	373	373	373	373	373	373
T_N_ (h)	186	**1**	186	186	186	186	186
T_A_ (h)	17	**1**	17	17	17	17	17
N_LIMP_	7	7	7	7	7	7	7
R_A_ (10^−4^ h^−1^)	10.230	10.230	10.230	10.230	10.230	10.230	10.230
R_NDiff_ (10^−4^ h^-1^)	0	0	0	0	0	0	0
R_ADiff_ (10^−4^ h^−1^)	2.591	2.591	**0**	2.591	2.591	2.591	2.591
P_G0toG1_ (h^−1^)	1	1	1	1	1	1	1
P_sleep_	0.179	0.179	0.179	**0.060**	0.179	0.179	0.179
P_sym_	0.090	0.090	0.090	0.090	**0.15**	0.090	0.090
α (10^−3^ Gy^−1^)	9.254	9.254	9.254	9.254	9.254	**150.000**	9.254
a/β (Gy)	3	3	3	3	3	3	3
OER	2.388	2.388	2.388	2.388	2.388	2.388	**1.00**

NOTE: for each column, the values that are different from those of Case-0, are indicated in bold font.

**TABLE 5:** Resulting tumor characteristics for the 6 different cases (7 including the baseline Case-0) tested with the imaging data of the patient considered for the qualitative study of the OncoSimulator. Patient 1 has been considered as reference.

	Doubling time (days)	Growth rate (10^−4^ h^−1^)	Necrotic and apoptotic cell fraction (%)	Growth fraction (%)	Stem cell fraction (%)	Differentiated cell fraction (%)	G0 cell fraction (%)
Case-0	484	0.597	0.447	1.057	0.040	97.176	1.768
Case-1	484	0.597	0	1.057	0.040	97.176	1.768
Case-2	485	0.596	0.009	0.208	0.008	99.444	0.348
Case-3	31	9.319	0.474	3.928	0.081	94.329	1.742
Case-4	30	9.561	0.523	4.071	0.203	90.22	5.708
Case-5	484	0.597	0.447	1.057	0.040	97.176	1.768
Case-6	484	0.597	0.447	1.057	0.040	97.176	1.768

The following observations can be made Figures 10, 11: Baseline Case-0 is a solution of patient 1 and refers to a scenario in which the tumor doubling time is approximately 500 days, the growth fraction is 1.3%, the initial tumor is basically composed of differentiated cells and the fraction of dead cells is very low. The scenario concerns a slowly developing tumor, a picture compatible with prostate cancer. In Case-1, we approach the minimally necrotic and apoptotic tumor scenario by minimizing *T_N_* and *T_A_*. The initial necrotic and apoptotic cell fraction is minimized to 0. The necrotic and apoptotic cell fraction is preserved to 0 through time, since necrotic and apoptotic cells are instantly eliminated and not accumulated. The rest of the tumor characteristics preserve the Case-0 reference behavior. In Case-2, the loss rate of differentiated cells is minimized. Again, the initial necrotic and apoptotic cell fraction is minimized to 0. As opposed to Case-1, the necrotic and apoptotic cell fraction is increased during therapy, since necrotic and apoptotic cells are accumulated over time. Moreover, the tumor composition changes. The differentiated cell population is increased. As a result, the stem and G0 cell fractions, as well as the growth fraction are decreased. Finally, the tumor volume reduction is decreased compared to the Case-0 reference behavior. In Case-3, we approach an aggressive tumor scenario by minimizing *P_sleep_*. Decreasing *P_sleep_* causes less cells to enter the dormant G0 phase and the number of proliferating cells is increased. As a result, mitosis happens more frequently and the number of stem cells is also increased. Since the proliferating cell population is the one that is primarily increased, the initial differentiated and G0 cell fractions are decreased. Finally, the tumor does not respond to therapy, grows exponentially and, as a result, all cell populations, even G0, differentiated, apoptotic and necrotic, increase. In Case-4, we approach an aggressive tumor scenario by maximizing *P_sym_*. Increasing Psym causes more stem cells being produced by the more frequent incidence of symmetric division. As a result, the proliferating and G0 cell populations are also increased and the initial differentiated cell fraction is decreased. Finally, the tumor does not respond to therapy, grows exponentially and, as a result, all cell populations, even differentiated, apoptotic and necrotic, increase. Case-5 is obtained by maximizing the values of parameters α, β of the LQ model. Increasing α and β causes the survival fraction to decrease and the cell kill ratio to increase. As a result, more living and non-differentiated cells are hit by therapy. The differentiated cell fraction is consequently increased and the growth fraction, stem cell and G0 cell fractions are decreased. Finally, a higher tumor shrinkage compared to Case-0 is observed. Case-6 is obtained by reducing the OER, which results in an increase in the cell kill ratio. Observations agree with Case-5. The previous observations are compatible with the scientific knowledge and intuition concerning the simulated biological processes, which confirms the correct operation of the OncoSimulator.

##### 3.1.2.2 Systematic sensitivity analysis

###### 3.1.2.2.1 Methods utilized

The one-factor-at-a-time sensitivity index (SI) was used to quantitatively rank the strength of the relationship between the output measure and the model parameters, employing a ±5% change in inputs (Hamby, 1994). Each input parameter was perturbed by ±5% from its baseline value, and the resulting percentage change in the output measure—final tumor volume, initial growth fraction, and initial growth rate—was recorded, with all other model parameters held at their baseline values. For input parameters with a strong influence on the output, where a ±5% variation results in biologically unrealistic tumor growth, a ±2.5% variation was considered.

The percentage changes in the output were then normalized to a ±1% variation in the input by dividing by the percentage change of the input, using the following formulas (Equations 8, 9):

$$SI_{+\%}=\frac{\left(Y_{base+\%}-Y_{base}\right)/Y_{base}}{\left(p_{i,base+\%}-p_{i,base}\right)/p_{i,base}}$$ (8)

$$SI_{‐\%}=\frac{\left(Y_{base-\%}-Y_{base}\right)/Y_{base}}{\left|p_{i,base-\%}-p_{i,base}\right|/p_{i,base}}$$ (9)

where *p*_*i,base*_: the baseline value of the *ith* parameter, *p*_*i,base*+(−)%_: the value of the *ith* parameter 5% or 2.5% above (or below) its baseline value, *Y*_*base*_: the output measure with all parameters at their baseline values, *Y*_*base*+(−)%_: the output measure with only the *ith* parameter set at 5% or 2.5% above (or below) its baseline value.

For simplification, all simulations assumed macroscopically homogeneous tumors, meaning that the model parameter values represent their spatial average throughout the tumor. The treatment schedule from Figure 4 was applied.

###### 3.1.2.2.2 Sensitivity analysis results

**FIGURE 12:**
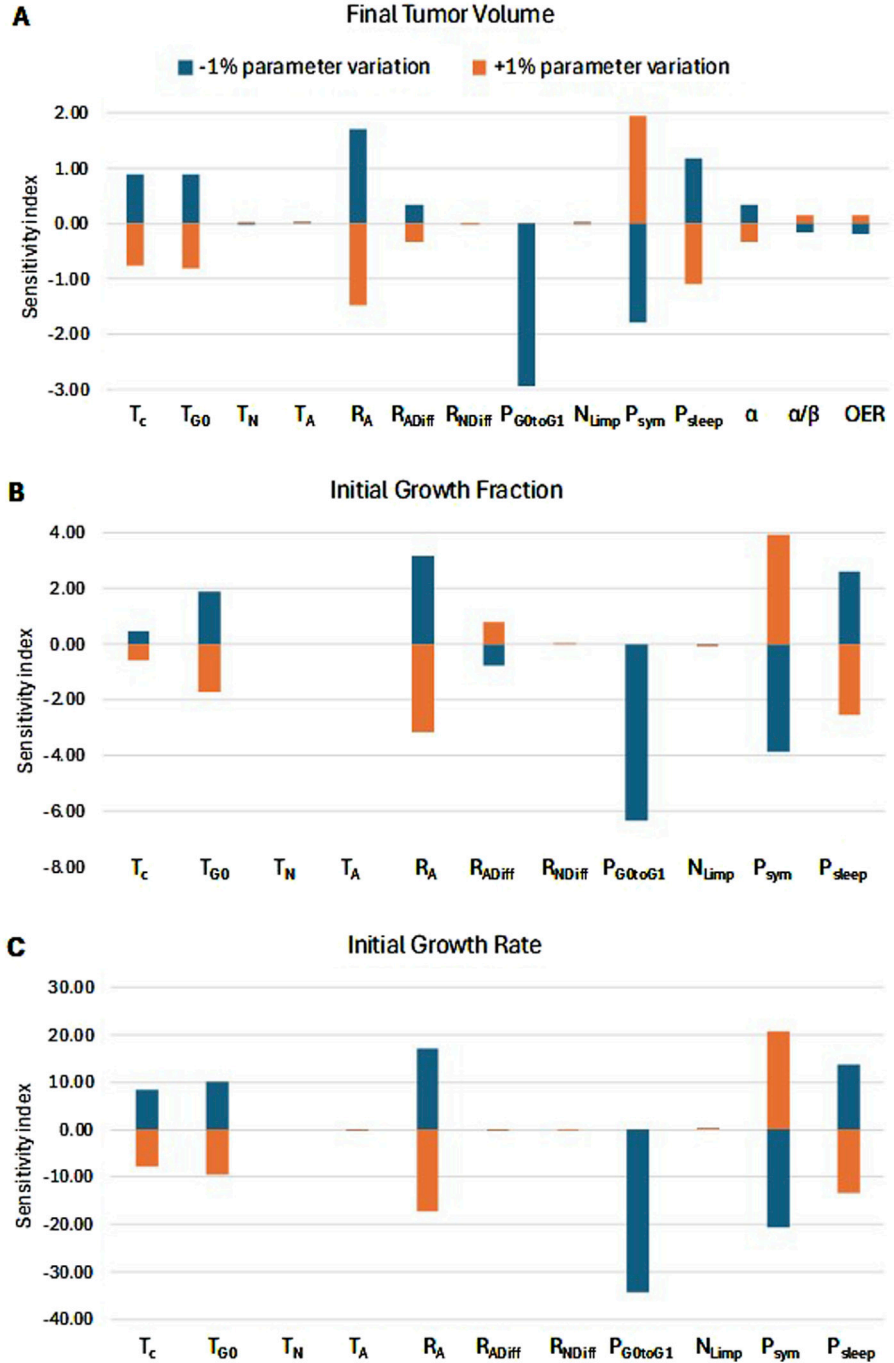
Sensitivity Analysis. Sorting of model parameters (see Table 1) based on their effects on: **(A)** radiation-induced tumor shrinkage, **(B)** growth fraction before radiation treatment, and **(C)** growth rate before radiation treatment. The sensitivity index for each input parameter is defined as the percentage change in the respective characteristic for every ±1% change in the input parameter. A positive correlation between an input parameter and the output measure is translated to a positive *SI*+% and a negative *SI*-%. On the other hand, a negative correlation between an input parameter and the output measure is translated to a negative *SI*+% and a positive *SI*-%. The highest the absolute value of *SI*, the strongest the correlation and, hence, the influence of the input parameter on the output measure. The radiation schedule of Figure 4 (Patient 1) has been simulated. The final tumor volume corresponds to day 119 after pretreatment MRI acquisition.

This section quantitatively ranks the sensitivity of the model parameters. Patient 1 has been considered as reference. Figure 12 depicts the percentage change in selected simulation outcomes resulting from a ±1% change in each model parameter around its baseline value. The simulation outcomes considered are the initial growth rate, the initial growth fraction, and the tumor volume at the time of post-treatment MRI acquisition (119 days after the first pre-treatment imaging study). The latter outcome is a standard measure of the response to radiotherapy in the clinical setting.

We observe that the percentage change in the output is asymmetric for changes above and below the baseline value of the parameters. The key biological mechanisms influencing the therapy outcome (Figure 12A) are:

a. The fraction of the dormant cells re-entering the cell cycle—*P*_*G0toG1*_ *.* This mechanism indicates how the oxygenation and nutrients’ availability status of the tumor plays a role in the model.
b. The oxygen and nutrients availability status of the tumor as represented mainly by the fraction of cells entering the dormant phase following mitosis—*P*_*sleep*_ *.*
c. The balance between the symmetric and asymmetric modes of stem cell division, reflecting intrinsic properties of stem cells and/or extrinsic controls from their microenvironment (represented by the fraction of stem cells that divide symmetrically—*P*_*sym*_).
d. The apoptosis rate of living stem and committed progenitor (LIMP) tumor cells—*R*_*A*_ *.*

These parameters significantly influence the tumor’s growth rate and growth fraction (Figures 12B, C). In contrast, the radiosensitivity parameters have a relatively small effect on tumor volume reduction. The local sensitivity is highly dependent on the baseline values of the parameters under investigation. The baseline values used correspond to Case 0 in Table 4, which represents a typical proliferation profile of prostate cancer with a low growth rate and growth fraction, and a negligible dead cell compartment.

## 4 Discussion

The work presented in this paper has provided the basics of the proposed model constitution, parameter handling and behavior, in relation to existing clinical and experimental knowledge in the prostate cancer radiotherapy domain. However, further *in silico* experimentation (e.g., more virtual tumors with differing characteristics to be considered and studied) and a thorough clinical validation and certification are needed in view of the envisaged clinical translation of the model. This will be the subject of future work. Specific aspects of the work presented are discussed below.

Regarding the mitotic potential of progenitor or LIMP tumor cells| (Figure 1), as a first approximation, LIMP cells are considered to stop differentiating after a specific number of divisions. Based on the results of the sensitivity analysis conducted (Figure 12), the model appears to be minimally sensitive on the exact number of mitoses performed by LIMP cells before becoming differentiated (NLIMP). Plausible average values of 7 up to 9 for NLIMP have been used and explored in the executions presented in this paper, including the clinical adaptation procedure.

As a first approximation, Psym; Psleep and PG0toG1 have been considered constant over the simulated time for the case of an apparently homogeneously oxygenated tumor. Their values reflect the average values of the corresponding parameters over time. However, if oxygenation inhomogeneities are detectable on the imaging data (e.g., MRI), algorithmic rules approximating the boundaries of the well and the poorly oxygenated regions of the tumor as time evolves, such as the ones proposed in (Stamatakos et al., 2002), could be used instead. The values of Psleep and PG0toG1 would then differ in the well and the poorly oxygenated regions. Since no inhomogeneities have been detectable on the imaging data (MRI slices) corresponding to the patient cases considered in this paper, oxygen biotransport has not been explicitly addressed. Nevertheless, average values of the related model parameters Psleep and PG0toG1 have been calculated as part of the solutions finding process during the proposed clinical adaptation methodology.

It is noted that as a first approximation, certain rather generic radiobiological parameter values for the prostate cancer treatment context addressed have been used in the present exploration. In a future more refined exploration of the model behavior, slightly better substantiated parameter values could be used. Approximations refer basically to the cancer cell density and the α/β value. Nevertheless, since the total *tumor volume reduction percentage* is one of the key outcomes of the exploration, no major changes are intuitively expected to arise if slightly different cell densities of cancer cells per cubic centimeter for prostate tumors are considered. Regarding the α/β value, the value of α/β = 3 has been considered. This value lies within the interval of the values of α/β that have been calculated for prostate cancer, e.g., by (Pedicini et al., 2013; Wang et al., 2003). In a future more refined analysis of the model behavior, slightly better substantiated parameter values could be used.

*In silico* studies related to *in vivo* radiosensitivity estimates in conjunction with the molecular profile of a patient appear to be good candidates for the identification of radioresistance and radiosensitivity profiles. To this end, machine learning can also be recruited in order to distinguish among radio sensitivity phenotypes and to quantify tumor cell response to treatment, based on whole genome analysis data, gene expression profiles and transcriptomic or proteomic signatures. Eventually, such studies could allow for the prediction of treatment outcome. If appropriate data is available (e.g., Ki-67 index and apoptotic index at diagnosis, imaging data before and during or shortly after therapy or radiosensitivity estimates based on molecular or genetic data), the OncoSimulator could predict the tumor evolution for the next few months. Such a prediction could support clinical decisions concerning modified treatments and/or the best time for surgery in the patient specific context. It is noted that Ki-67 is an excellent marker to determine the growth fraction (GF) of a given cell population (Wikipedia, 2024). Therefore, availability of Ki-67 can lead to a credible estimation of growth fraction (GF), of which the use is delineated, *inter alia*, in Section 3.1.1.

Regarding the limiting assumption of tumor free growth within the substantially deformable soft organ of prostate (Section 1, Introduction), the model could be improved regarding its mechanical (shape) related aspects, by combining our discrete entity–discrete event approach with the Finite Elements method for biomechanics. Such an approach has already been applied to the case of glioblastoma growth simulation by Bauer et al. (2012).

As far as model parametrization and clinical validation are concerned, a large number of multiscale data series in the context of both retrospective and prospective clinical studies is currently being collected. These include *inter alia* data originating from our team’s work in the context of the German clinical study HypoFocal-SBRT (German Clinical Trial Register, 2024) and the work published in Marinescu et al. (2022). It should be made clear, however, that the present paper has presented the initial steps of the work towards the development of a prostate cancer OncoSimulator. Its aim has been to showcase the feasibility, the fundamental design and the qualitative behavior of the model.

Regarding the side effects of radiation therapy on normal tissues, including gastrointestinal and genito-urinary sequels that shape an important part of the individual preferences of the patient, these can be addressed as follows. An extended discretizing mesh is superimposed on the anatomic region of interest. The latter, apart from the tumor foci, also covers the adjacent irradiated normal tissues. Following calculation of the expected absorbed radiation dose at each geometrical cell of the mesh, the bio-simulation part begins. Application of the key biological, physiological, physical, chemical and biomechanical laws and/or rules, which collectively represent the overarching principle of *homeostasis* at each geometrical cell filled by specific normal tissue–and eventually affected by its neighboring geometrical cells - leads to the prediction of a crucial part of the expected side effects of treatment (acute and late toxicity). This can be implemented in a way quite similar to the one adopted above for the case of tumor response to treatment. Such an approach has already been applied by the authors’ team to the case of glioblastoma multiforme treated by radiotherapeutic schemes (Antipas et al., 2007). By comparing the expected response of the tumor with that of the irradiated normal tissues to each candidate radiotherapeutic scheme and by also taking into account the patient preferences regarding both the irradiation procedure (e.g., number of irradiation sessions per week and total irradiation duration) and the expected treatment side effects (e.g., incontinence, impotence), a more advanced level of personalized treatment optimization could be achieved. Such an approach is, however, highly demanding in terms of modelling and simulation effort; therefore, this will be the subject of future work.

## 5 Conclusion

A multiscale mechanistic computational model of prostate tumor growth and response to radiotherapy has been presented as a first step towards the development of a prostate cancer digital twin (OncoSimulator). Special emphasis has been put on the histological constitution of the tumor and its temporal response to radiotherapeutic treatment. Following technical verification, an adaptation to clinical data approach has been delineated and an initial exploration of its potential has been outlined. In addition, a parametric and sensitivity analysis, which has revealed the impact of particular model parameters on the overall model behavior, has been performed. A qualitative agreement of the proposed model behavior with published experimental and clinical knowledge and data for two patients has set up the basis for the next steps towards its thorough clinical validation and its eventual clinical translation. It is pointed out, however, that the content of this paper is of preliminary nature. Its outcome has been the demonstration of the feasibility, the basic design and the core behavior of the model. Further data is being collected in order to enhance model parametrization and conduct a rigorous clinical validation. The envisaged digital twin or OncoSimulator, to be built around the model presented—provided that both clinical validation and regulatory certification are favorable—is to be exploited for both patient individualized treatment and *in silico* clinical trials in the context of prostate cancer treated with radiation therapy.

## Data Availability

The original contributions presented in the study are included in the article/supplementary material, further inquiries can be directed to the corresponding author.
